# BABEL enables cross-modality translation between multiomic profiles at single-cell resolution

**DOI:** 10.1073/pnas.2023070118

**Published:** 2021-04-07

**Authors:** Kevin E. Wu, Kathryn E. Yost, Howard Y. Chang, James Zou

**Affiliations:** ^a^Department of Computer Science, Stanford University, Stanford, CA 94305;; ^b^Department of Biomedical Data Science, Stanford University School of Medicine, Stanford, CA 94305;; ^c^Center for Personal and Dynamic Regulomes, Stanford University School of Medicine, Stanford, CA 94305;; ^d^HHMI, Stanford University School of Medicine, Stanford, CA 94305

**Keywords:** single-cell analysis, multiomics, deep learning, gene regulation

## Abstract

Simultaneous profiling of multiomic modalities within a single cell is a grand challenge for single-cell biology. While there have been impressive technical innovations demonstrating feasibility—for example, generating paired measurements of single-cell transcriptome (single-cell RNA sequencing [scRNA-seq]) and chromatin accessibility (single-cell assay for transposase-accessible chromatin using sequencing [scATAC-seq])—widespread application of joint profiling is challenging due to its experimental complexity, noise, and cost. Here, we introduce BABEL, a deep learning method that translates between the transcriptome and chromatin profiles of a single cell. Leveraging an interoperable neural network model, BABEL can predict single-cell expression directly from a cell’s scATAC-seq and vice versa after training on relevant data. This makes it possible to computationally synthesize paired multiomic measurements when only one modality is experimentally available. Across several paired single-cell ATAC and gene expression datasets in human and mouse, we validate that BABEL accurately translates between these modalities for individual cells. BABEL also generalizes well to cell types within new biological contexts not seen during training. Starting from scATAC-seq of patient-derived basal cell carcinoma (BCC), BABEL generated single-cell expression that enabled fine-grained classification of complex cell states, despite having never seen BCC data. These predictions are comparable to analyses of experimental BCC scRNA-seq data for diverse cell types related to BABEL’s training data. We further show that BABEL can incorporate additional single-cell data modalities, such as protein epitope profiling, thus enabling translation across chromatin, RNA, and protein. BABEL offers a powerful approach for data exploration and hypothesis generation.

## Significance

Simultaneous measurement of the DNA, RNA, and proteins of single cells can lead to important new insights but is experimentally challenging. This work introduces a deep learning algorithm that flexibly translates between chromatin, RNA, and protein profiles in single cells. This makes it possible to computationally synthesize matched multiomic measurements when only one modality is experimentally available. This algorithm complements experimental advances to efficiently achieve single-cell multiomic insight.

**Fig. 1.**
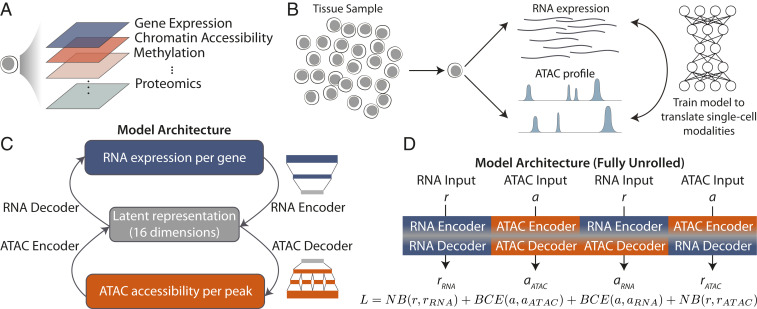
Overview of cross-modality single-cell translation with BABEL. (*A*) Advances in single-cell sequencing technology have enabled a myriad of single-cell modalities, ranging from gene expression to proteomics, as well as technologies jointly profiling combinations thereof. However, multimodal profiling also presents challenges such as increased complexity, noise, and cost. (*B*) We investigate an alternative approach to single-cell multiomic profiling by using machine learning to translate between single-cell omics measurements, thus enabling the inference of unmeasured modalities. For our study, we focus on RNA expression and ATAC chromatin accessibility. *C* shows BABEL’s modeling strategy: Two encoders project ATAC (orange) and RNA (blue) into a shared latent space (gray), and two decoders take points in the latent space and infer their corresponding ATAC or RNA profiles. These encoders and decoders are interoperable by design, projecting into and out of the same latent representation. The RNA networks use a series of fully connected layers (blue schematic), while the ATAC networks break into subconnections that limit the model to learning predominantly intrachromosomal weights (orange schematic), which greatly reduces model complexity. *D* summarizes the four possible combinations of the encoders and decoders in our network and shows the joint loss function (colors indicate data modality). We train the model by passing each paired ATAC/gene expression measurement through every combination of encoders and decoders, and our loss L ensures that all four subnetworks work together to produce accurate translations.

Single-cell technologies have made it possible to precisely characterize cellular state using diverse modalities ranging from gene expression and chromatin accessibility, to proteomics and methylation (Fig. 1*A*) (1). Such fine-grained measurements provide much more information beyond the average bulk state of a tissue sample and have enabled novel insights into complex biological systems. However, a notable limitation of standard single-cell technologies is that they only capture one measurement modality (e.g., only RNA sequencing [RNA-seq] or only chromatin accessibility [assay for transposase-accessible chromatin using sequencing, ATAC-seq]) for each cell. This loses critical information about how different layers of genomic regulation interact within individual cells.

More recently, multiomic single-cell methods jointly profiling multiple modalities within the same cell have emerged (1, 2). For example, SNARE-seq (single-nucleus chromatin accessibility and mRNA expression sequencing) and sci-CAR (single-cell combinatorial indexing profiling chromatin accessibility and mRNA) combine chromatin accessibility with RNA gene expression measurements (3, 4), CITE-seq (cellular indexing of transcriptomes and epitopes by sequencing) enables joint quantification of RNA expression and protein markers (5), and Pi-ATAC (protein-indexed assay of transposase accessible chromatin) and ASAP-seq (ATAC with select antigen profiling) merge epigenomic and protein measurements (6, 7). These methods’ paired measurements have helped researchers gain a more comprehensive understanding of how different cellular mechanisms interact. As an example, coassays of accessibility and expression specifically identified distal *cis*-regulatory elements for genes that do not exhibit clear cell type-specific promoter accessibility (4).

However, these joint single-cell methods face challenges of their own. Single-cell multiomics methods often require additional precautions when preserving or isolating cells in order to effectively capture a diverse range of molecules, with RNA often being the most difficult to handle and store (8). Imperfections in this step can lead to increased noise and drop-out in the resulting data. As noise and sparsity are already substantial hurdles in analyzing single-cell data (9), this can make extracting reliable insights from multiomic single-cell data particularly challenging. Beyond technical feasibility, the increased costs of these multiomic experiments can also limit the scale at which they can be performed (2). With these challenges, it may not always be possible or practical to experimentally jointly profile single cells, which motivates the question of how we can extract the most information from samples where only one modality can be captured.

We develop BABEL, a deep learning algorithm that computationally generates, from a single measured modality, other multiomic modalities in the same single cell. This enables researchers to perform downstream multiomic analysis at single-cell resolution as if joint profiling data had been collected. This approach is analogous to translating sentences between languages with different grammatical and syntactic structures. BABEL can accurately infer transcriptome-wide single-cell RNA profiles from genome-wide single-cell ATAC profiles, and vice versa (Fig. 1*B*). We focus on RNA and ATAC in this work, because experimental methods for jointly profiling these modalities are more advanced and have the most data available.

After training BABEL on cells with jointly profiled chromatin accessibility and transcriptomic measurements, we first demonstrate that BABEL performs well on test cell types and tissues distinct from the cell population used for training. We then show that BABEL can be applied to single-modality single-cell experiments, inferring high-quality cross-domain data that can be analyzed to produce similar conclusions compared to carrying out an entirely separate experiment. We then successfully apply BABEL to analyzing patient basal cell carcinoma (BCC) samples profiled using single-cell ATAC sequencing (scATAC-seq) (10). Although this is particularly challenging due to the heterogeneity of tumor microenvironments, BABEL’s predictions are concordant with previous findings and help uncover additional information compared to previous methods. Throughout our analyses, we find that BABEL’s predictions are consistently driven by individual cell signatures, rather than bulk approximations. Finally, as a proof-of-concept demonstrating BABEL’s versatility and extensibility, we show that BABEL can predict single-cell epitope profiles from scATAC-seq, enabling matched single-cell chromatin, RNA, and epitope analysis even though such data are not yet experimentally available.

Several prior works have applied deep learning methods to single-cell data. Many of these focus on developing models to denoise single-cell RNA-seq (scRNA-seq) data. Examples of these methods include DeepCountAutoencoder, which trains an autoencoder for denoising scRNA-seq data (11), and SAUCIE, which uses a similar autoencoder along with clever regularization to denoise, batch-correct, and cluster scRNA-seq data (12). Other approaches, such as scVI, apply generative modeling to develop models that facilitate downstream analyses like batch correction and differential expression (13). scATAC-seq data has been modeled with machine learning approaches as well, with works like SCALE using autoencoders to learn latent representations conducive to clustering (14). Deep learning methods for multiomic data have also been studied, but these prior works generally did not have access to large-scale paired measurements, which motivated complex techniques to align latent representations (15–18) or constrained these works to bulk measurements (19). More recently, new experimental techniques for generating paired single-cell data have enabled more streamlined multimodal modeling of protein epitopes and transcriptomics (20) as well as of physiological profiles and transcriptomics (21). BABEL builds off these prior works while introducing strategies for more efficient model architectures and latent space learning. BABEL is a method that accurately and robustly translates between gene expression and chromatin accessibility profiles for individual cells.

BABEL addresses a different problem from typical multiomic data integration. Prior methods such as iCluster (22), Seurat (23), ArchR (24), MAESTRO (25), MATCHER (26), and various matrix factorization approaches (27–29) excel at data integration, where they take two (typically unpaired) data modalities that have already been measured and compute joint clustering, identify cell-to-cell mappings, and infer cross-domain interactions. BABEL’s goal is to build a generalizable model that takes only one of these modalities and infers the other, thus enabling multiomic analysis. BABEL provides a powerful tool for hypothesis generation and exploratory analysis, especially in instances where the original sample might no longer be available, cannot be reacquired, or does not contain all necessary molecules, as is often the case for clinical or archival samples (30).

## Results

### BABEL Architecture and Design.

BABEL consists of four modular neural networks as subcomponents (Fig. 1*C*). Two encoder networks are trained to project either RNA or ATAC profiles into a single, shared 16-dimensional latent representation. Similarly, two decoder neural networks are trained to take points in this shared latent representation and infer their corresponding RNA or ATAC profiles. This shared latent space simultaneously summarizes the epigenetic and transcriptomic profiles of a cell and can be thought of as an abstract, integrated representation of cellular state. This latent space also serves as an intentionally low-dimensional “information bottleneck” to encourage the model to capture major cellular variation, rather than potentially spurious deviations (*SI Appendix*, *Supplementary Note* and Table S1). Our goal is then to learn encoder models that project single-cell profiles into this cellular state latent space, along with decoder models that can infer observed phenotypes from this same latent cellular representation.

While a standard autoencoder maps one data modality onto itself (e.g., scATAC-seq to scATAC-seq), BABEL maps across multiple modalities (e.g., it uses the same ATAC encoder to map ATAC to ATAC and ATAC to RNA). This enables the model to be more flexible and efficient. The networks responsible for RNA encoding and decoding consist of fully connected layers that project a continuous, transcriptome-wide expression vector to and from the latent cellular representation (Fig. 1*C*). The networks responsible for encoding and decoding a binarized genome-wide ATAC chromatin accessibility signal are also composed of fully connected layers, but we leverage the insight that most chromatin accessibility interactions occur at an intrachromosomal level (31) to prune the majority of interchromosomal connections (Fig. 1*C*). This approach substantially reduces the parameter space and helps the model avoid spurious correlations (*Methods* and *SI Appendix*, *Supplementary Note*). We binarize all ATAC measurements throughout this work—any peak with nonzero signal is set to “1” and is otherwise “0.” BABEL then predicts the probability that a peak is active (i.e., 1). This approach for learning on scATAC-seq data has been used in prior scATAC-seq models (14) and improves the quality of BABEL’s ATAC predictions.

BABEL is trained using a loss function that requires both encoders to be interoperable with either decoder. This is expressed by enumerating all four possible compositions of our two encoders and two decoders and simultaneously training all four of these “paths” through our model to produce correct outputs using four corresponding loss terms (Fig. 1*D*). Under this formulation, the ATAC encoder’s latent output must be consumable by both the ATAC decoder and RNA decoder while producing correct outputs for both modalities, and the same goes for RNA encoder. Similarly, the ATAC and RNA decoders must understand the latent representation generated by either ATAC or RNA encoder. This interoperability constraint leverages paired data to learn a single, unified latent space that embeds multimodal cell state without explicit latent space alignment, while improving BABEL’s ability to generalize to unseen cell types (*SI Appendix*, *Supplementary Notes* and Table S2). To evaluate the correctness of inferred RNA values (whether these inferences were generated from an ATAC or RNA input), we use a negative binomial (NB) loss, which has seen success in prior works imputing and denoising single-cell expression (11, 13). Notably, this loss encourages BABEL to estimate the denoised single-cell expression, rather than the noisy experimental counts (11). To evaluate the correctness of inferred ATAC values, we use a binary cross entropy (BCE) loss—a natural cost function for binary predictions used in prior deep learning models for scATAC-seq data (14). After training, BABEL can translate between a continuous vector representing an individual cell’s (denoised) transcriptome-wide gene expression spanning 34,861 genes, and a probability vector describing genome-wide ATAC accessibility profiles spanning 223,897 high-resolution peaks with mean and median widths of 796 and 573 base pairs.

### BABEL Performs Cross-Domain Translation with High Accuracy.

**Fig. 2.**
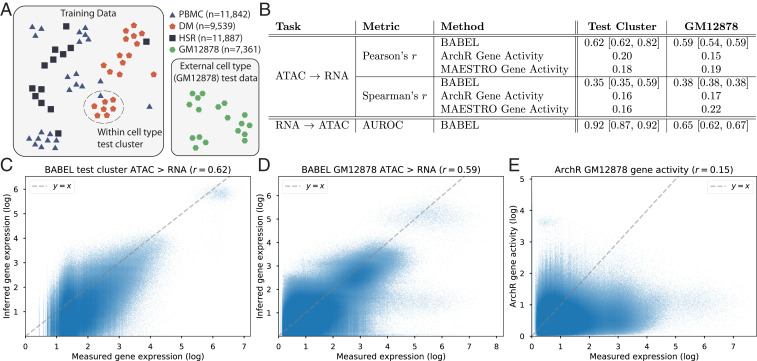
Summary of train and test data, and BABEL’s performance. (*A*) We train BABEL using PBMC, DM, and HSR cells with jointly profiled ATAC and RNA measurements. Data from these three are pooled and clustered (“Training Data” box), with one cluster reserved as a test cluster. Splitting by cluster reduces similarity between train and test data. Moreover, we exclude a set of jointly profiled GM12878 cells from all training purposes, used only for model evaluation (“External cell type” box). (*B*) BABEL’s performance on the test cluster and external GM12878 evaluation set, with cross-validation performance ranges in brackets (*SI Appendix*, Tables S3 and S4). To contextualize BABEL’s ATAC to RNA performance, we evaluate comparable gene activity score estimation methods from ArchR and MAESTRO. (*C*) Density scatterplot showing the expression of each gene in each cell within the test cluster (*x* axis represents empirical expression; *y* axis represents BABEL’s inferred expression). BABEL exhibits a strong correlation of 0.62 on the test cluster. (*D*) Similarly shows BABEL’s inferences on the external GM12878 evaluation set. (*E*) ArchR gene activity scores for these GM12878 cells, which are less accurate than BABEL’s inferences.

We train BABEL using single-cell multiomic data jointly profiling ATAC chromatin accessibility and RNA gene expression generated on 10x Genomics’ multiomic platform, which starts from single nuclei (*Methods*). These data span cells collected from several human primary cell types and transformed cell lines: peripheral blood mononuclear cells (PBMCs), colon adenocarcinoma COLO-320DM (DM) cells, colorectal adenocarcinoma COLO-320HSR (HSR) cells, and lymphoblastoid GM12878 cells. We pool and cluster the PBMC, DM, and HSR cells together, reserving one cluster for validation (*n* = 2,881 cells) and one cluster for test (*n* = 1,979 cells), with the remaining cells (*n* = 28,408) being used for training (Fig. 2*A* and *SI Appendix*, Table S3). Although BABEL itself is agnostic of cluster identity, cluster-based data splits reduce similarity between data splits, challenging the model to generalize to new cell populations. Jointly profiled GM12878 cells are held out from any training purposes, serving as a measure of generalization even more challenging than the test cluster.

BABEL achieves strong performance for cross-domain inference on all test data. Inferring RNA expression from ATAC accessibility, it achieves a Pearson correlation of 0.62 and a Spearman’s correlation of 0.35 (Fig. 2 *B* and *C*). Inferring ATAC from RNA on this same test cluster, BABEL achieves an area under the receiver operating characteristic (AUROC) of 0.92. We use different metrics for evaluating ATAC and RNA predictions due to the binary versus continuous nature of these modalities. BABEL’s performance is consistent across cluster-based cross-validation as well, exhibiting ATAC to RNA Pearson’s correlations ranging from 0.62 to 0.82 with a median of 0.80, and RNA to ATAC AUROCs ranging from 0.87 to 0.92 with a median of 0.87 (Fig. 2*B* and *SI Appendix*, Table S3).

We evaluated *k*-nearest-neighbor (KNN) models to establish performance baselines for these translation tasks, using the same cluster-based data splits. A KNN model takes each query point, identifies the (*k* = 10) most similar cells in the training set, and computes an average of those cells (see *Methods* for more details). KNN achieves a Pearson correlation of 0.27 when inferring RNA from ATAC on the aforementioned test set, and an AUROC of 0.84 when inferring ATAC from RNA. BABEL significantly outperforms both of these baselines.

To quantify BABEL’s ability to generalize to a different cell type, we applied BABEL trained on PBMC, DM, and HSR data to paired single-cell ATAC/gene expression data profiling GM12878, without any tuning or modification. This constitutes a challenging test since the GM12878 lymphoblastoid cell line exhibits substantial differences from the three cell types used for training. BABEL generalizes well to the GM12878 external evaluation set with an ATAC to RNA Pearson correlation of 0.59, Spearman correlation of 0.38, and an RNA to ATAC AUROC of 0.65 (Fig. 2 *B* and *D*). BABEL’s performance on GM12878 is also robust across cross-validation, where the training and validation clusters shift, and the five resultant models are evaluated on the same GM12878 evaluation set (Fig. 2*B* and *SI Appendix*, Table S4). Performance is similar for intradomain translations (i.e., inferring RNA output from RNA input and ATAC from ATAC input), which further validates BABEL’s subcomponent networks (*SI Appendix*, Tables S3 and S4).

Since one key objective of BABEL is to infer RNA gene expression from ATAC accessibility, existing tools that infer gene activity scores from ATAC data offer natural benchmarks. Like BABEL, these tools attempt to estimate the gene expression corresponding to an ATAC profile, drawing from observations and patterns present in prior data. Critically, while BABEL uses machine learning techniques to train a model, these existing tools use expert knowledge to hand-craft formulas based on prior observations that accessibility near a gene correlates with its expression. We specifically evaluated the approaches implemented by state-of-the-art scATAC-seq analysis suites ArchR (24) and MAESTRO (25). Evaluating the ArchR and MAESTRO gene activity scores on the GM12878 paired data yields Pearson’s correlations of 0.15 and 0.19, respectively, compared to BABEL’s 0.59 (Fig. 2 *B* and *E*). Since GM12878 was never used for training BABEL, nor was it the singular benchmark for developing gene activity scores, this represents a fair comparison where all methods are given similar input and are asked to generate similar outputs. For benchmarking the opposite RNA to ATAC translation, we are not aware of any prior methods that perform single-cell prediction. BIRD is a recently developed relevant method, although it is trained to make cluster-aggregated predictions of ATAC signals instead (32, 33), and consequently may be less flexible when applied to cell types it has not seen before. BABEL compares favorably to BIRD as well (*SI Appendix*, Fig. S1).

We additionally investigated how BABEL could perform on nonhuman data. We trained a separate version of BABEL on paired single-cell ATAC/gene expression data from the adult mouse cerebral cortex, generated via the SNARE-seq joint profiling protocol, which like the 10x multiomic data, profiles individual nuclei (3). On the held-out test cluster, BABEL achieves an ATAC to RNA Pearson correlation of 0.55, and an RNA to ATAC AUROC of 0.80 (*SI Appendix*, Fig. S2). As a second, independent mouse experiment, we trained BABEL on paired single-cell ATAC/gene expression data profiling mouse skin, generated via the SHARE-seq protocol (34). Unlike previous datasets, this SHARE-seq dataset is generated from single cells, rather than nuclei. On the held-out test cluster, BABEL exhibits an ATAC to RNA Pearson correlation of 0.53, and an RNA to ATAC AUROC of 0.80 (*SI Appendix*, Fig. S3). All of these values are similar to those we observed on the human dataset. Together, these results demonstrate that BABEL is applicable across different species and can be successfully trained using data generated by a variety of experimental protocols.

### BABEL Cross-Modality Inference Captures Empirically Validated Cell States.

**Fig. 3.**
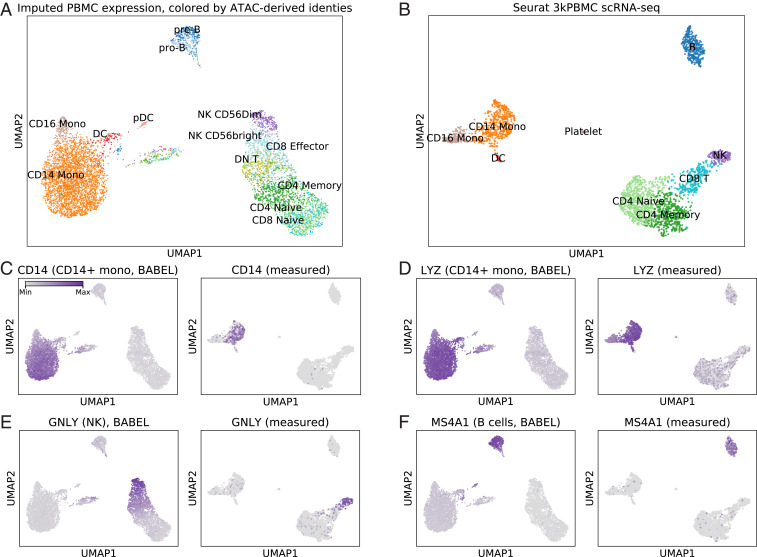
BABEL’s ATAC to RNA translation closely matches empirical results on unpaired PBMCs. (*A*) UMAP visualization of single-cell expression profiles imputed by BABEL from scATAC-seq, colored by ATAC-derived cell type identities. (*B*) UMAP visualization and cell types in empirical PBMC scRNA-seq for comparison. These two plots exhibit highly concordant global structure (i.e., both show three main cell groups) as well as very similar inter-cell type relationships. *C* highlights the expression of *CD14* (a marker for CD14^+^ monocytes) within BABEL’s inferred gene expression (*Left*) and the empirical measurements (*Right*). In both cases, *CD14* expression is highly correlated with CD14^+^ monocytes, as expected. (*D*) Similarly illustrates expression of *LYZ*, which is also a marker for CD14^+^ monocytes. *E* and *F* highlight expression of *GNLY* and *MS4A1*, which are markers for NK and B cells, respectively.

It is especially interesting to apply BABEL in settings where we do not have paired measurements, but where BABEL has been trained on reasonably similar cell types. This can enable exploratory analyses and hypothesis generation on new data via computationally imputed paired measurements. As a case study for this application, we reconstruct expression profiles for a set of healthy PBMC cells profiled using scATAC-seq. These unpaired data were generated using a different experimental protocol than was used to generate BABEL’s paired training data, and thus exhibit markedly different noise patterns that we do not explicitly adjust for (*SI Appendix*, Fig. S4). We use the pretrained BABEL to impute transcriptome-wide RNA expression signatures for each cell. We then apply standard preprocessing (size normalization and log-transformation) to BABEL’s inferred single-cell expression, and visualize the cells using the uniform manifold approximation and projection (UMAP) algorithm (35, 36) applied to their imputed expression signatures. We color each cell by its original ATAC-based cell type (*SI Appendix*, Fig. S5*A*) to visualize how well these are retained in BABEL’s imputed RNA profiles (Fig. 3*A*).

For comparison, we include a parallel clustering and analysis of empirical single-cell expression in healthy PBMCs (Fig. 3*B*). We find many similarities between our imputed visualization and this empirical visualization. There are three major clusters in both the imputed and empirical data. One major cluster corresponds to B cells (Fig. 3 *A* and *B*, *Top*), one consists of CD14^+^/CD16^+^ monocytes and dendritic cells (*Left*), and one primarily contains CD4, CD8, and natural killer (NK) cells (*Bottom Right*). These results suggest that BABEL’s imputed single-cell gene expression retains much of the empirical global gene expression patterns and relationships. Further analysis also reveals that this overall concordance is consistent across variants of BABEL trained on different cross-validation folds (*SI Appendix*, Table S5) and is also recapitulated in BABEL’s latent representation (*SI Appendix*, Fig. S5). This consistency suggests that BABEL can recognize complex relationships between cells in its input—despite variable noise patterns—and leverages these biological relationships when generating latent representations and predicting transcriptomic profiles. This property helps BABEL generalize to more contexts (*SI Appendix*, *Supplementary Note*) and suggests that BABEL’s latent representation could be a potentially interesting basis for downstream analyses like clustering or lineage tracing.

In addition to examining cell cluster concordance, we evaluate how well BABEL imputes expression of well-known marker genes for specific cell types. *CD14* is a canonical marker for CD14^+^ monocytes. Coloring each cell in BABEL’s expression UMAP by its imputed *CD14* expression, we find that *CD14* expression coincides very well with CD14^+^ monocyte cells (as identified via standard scATAC-seq analysis methods; Fig. 3*C*, *Left*). As expected, experimentally measured *CD14* expression overlaps nearly perfectly with CD14^+^ cells as well (Fig. 3 *C*, *Right*). Performing a similar comparison for *LYZ*, another well-known marker for CD14^+^ cells, we see that BABEL likewise reproduces experimentally validated expression distributions (Fig. 3*D*). Such concordance extends to other cell types as well. Examining the expression of *GNLY*, a marker for NK cells (Fig. 3*E*), and *MS4A1*, which corresponds to B cells (Fig. 3*F*), we consistently see that BABEL’s imputed marker gene expression matches the correct cell types just as the empirically measured expression does. This shows that BABEL is not just predicting average expression of genes across all cells regardless of ATAC profile but is performing highly specific expression inference for individual cells. These results suggest that BABEL can facilitate downstream cell type analysis by computationally generating missing data modalities. Although BABEL’s strong performance here benefits from having seen similar PBMC cells in its training set (*SI Appendix*, *Supplementary Note* and Fig. S6), generalizing to this external PBMC dataset is nonetheless challenging due to aforementioned differences in experimental protocols used to generate the data.

We also performed the opposite study, taking a set of PBMC cells profiled using unpaired scRNA-seq and inferring their genome-wide chromatin accessibility signatures. Having previously explored the performance of BABEL’s RNA to ATAC predictions, we now assess the usefulness of these predictions by investigating whether they recover meaningful biological signals. We focus on a cluster of NK cells (overexpressing *GNLY*), as these cells appear to have the most unambiguous transcriptomic signature. Among BABEL’s accessibility predictions, we identified the 10 most predictive peaks for these NK cells and determined the nearest annotated transcribed region for each resultant peak (see *Methods* for more details). This highlights known NK pathways and proposes potential unstudied regulatory interactions. BABEL’s predictions implicate a region proximal to *CD96*, a well-characterized regulator of NK cell function (37, 38). BABEL’s predictions also highlight *LINC01550*, a long noncoding RNA identified as an immune-related oncogenic biomarker (39), and *FOXP1*, a transcriptional regulator in T cell development (40). This case study shows that BABEL can make reasonable expression-to-accessibility predictions that may be useful for hypothesis generation.

### BABEL Can Generate New Insights for Patient Samples.

We next apply BABEL to scATAC-seq data acquired from BCC tumors to investigate its application on challenging patient samples without any additional fine-tuning or training. These samples, acquired from seven BCC patients, represent malignant, stromal, and immune cells present within the tumor microenvironment both before and after anti-programmed cell death protein 1 (PD-1) immunotherapy, and were profiled using scATAC-seq to generate chromatin accessibility profiles for 37,818 cells (10). These cells were originally analyzed using standard scATAC-seq methods by calculating gene activity scores with Cicero (41) and using these scores to label cell types and infer lineages.

**Fig. 4.**
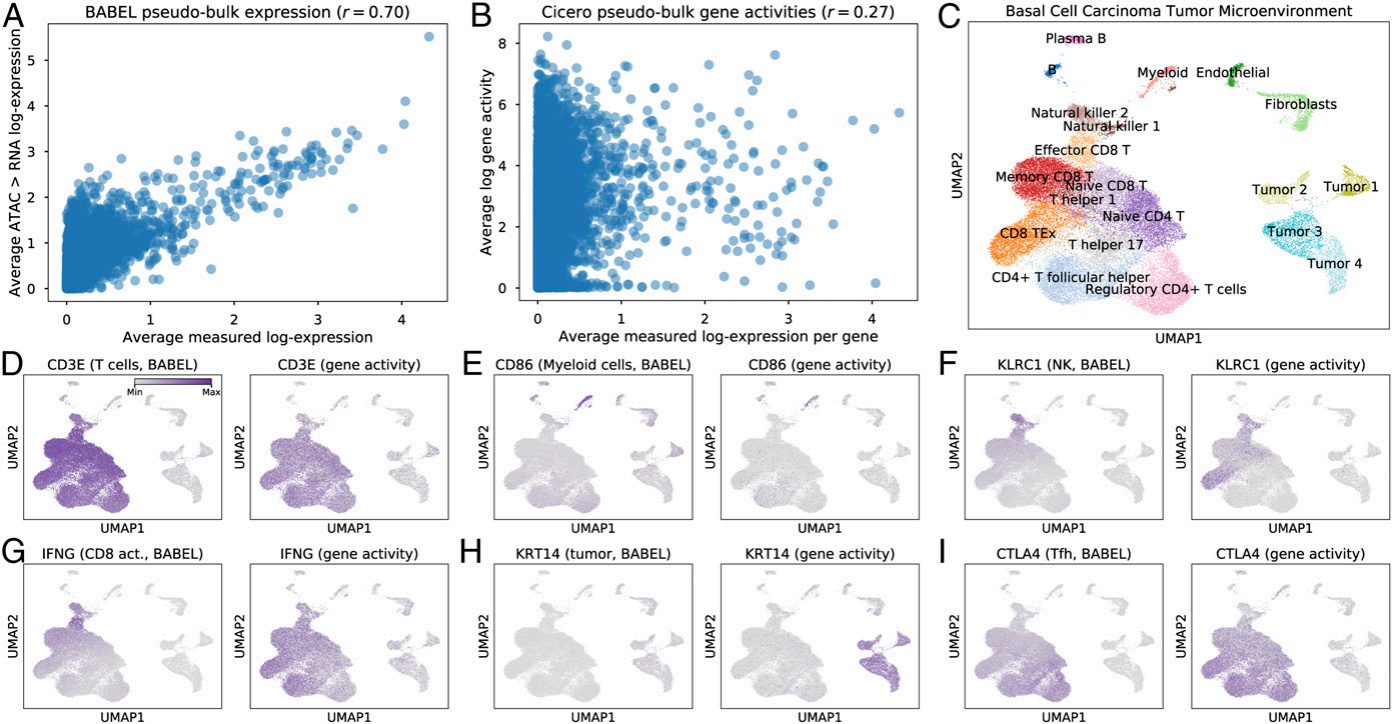
BABEL makes accurate predictions on clinical basal cell carcinoma (BCC) scATAC-seq samples and generates new interpretations. (*A*) Correlation of transcriptome-wide pseudobulk expression: *x* axis represents expression within a tissue-matched scRNA-seq study averaged across cells; *y* axis represents BABEL’s predicted single-cell expression from scATAC-seq data, also in pseudobulk. The strong correlation, especially compared to a similar plot for gene activity scores (*B*), suggests that BABEL generalizes well to patient cancer samples. (*C*) ATAC-based UMAP visualization of these BCC cells. Produced using data from ref. 10. We use this projection as a scaffold to visualize BABEL’s imputed single-cell RNA expression. *D* highlights BABEL’s imputed expression of *CD3E* (*Left*), a T cell marker, compared to gene activity scores (*Right*). BABEL recapitulates expected expression of *CD3E* here, as well as for the myeloid marker *CD86* (*E*). For NK marker *KLRC1* (*F*) and CD8^+^ activated T cell marker *IFNG* (*G*), BABEL predicts more localized, specific expression than gene activity scores. However, for genes that have little to no presence in the training data such as *KRT14*, BABEL shows weaker performance (*H*). BABEL also lets us expand on prior conclusions, predicting more distinct overexpression of immunosuppressive genes like *CTLA4* (*I*) in the Tfh cluster (*SI Appendix*, Fig. S8), strengthening these cells’ reported similarity to exhausted CD8^+^ cells.

A superset of these BCC samples has also been studied in a separate, unpaired experiment using scRNA-seq profiling (42), which enables evaluation of aggregate concordance metrics. Specifically, we leverage the intuition that by averaging across all cells in a single-cell experiment, the resulting per-gene pseudobulk expression values should be comparable across related experiments. We compared pseudobulk RNA expression profiles derived from BABEL’s inferred single-cell expression against empirical pseudobulk from the corresponding patients’ tissue-matched scRNA-seq, and found good agreement (Pearson’s *r* = 0.70; Fig. 4*A*). This concordance also holds when we examine specific cell types, rather than the global population (*SI Appendix*, Fig. S7). Furthermore, the mismatch between BABEL prediction and empirical scRNA-seq tended to manifest as increased predicted expression for genes with low observed expression, which could have been experimentally underreported due to drop-outs. For context, we also calculated the pseudobulk correlation of Cicero’s gene activity scores against the empirical pseudobulk and observed a weaker correlation of 0.27 (Fig. 4*B*). Overall, BABEL’s predicted RNA expression profiles based on BCC scATAC-seq are robust. More generally, we propose that aggregate concordance metrics like those described here can serve as simple checks when applying BABEL to a new sample. Comparing BABEL’s aggregated predictions to known bulk signatures and observing a high correlation would indicate that BABEL is likely making reasonable predictions, whereas low correlation might suggest BABEL may not generalize well on a particular sample.

We overlay BABEL’s predicted expression on the ATAC-based UMAP projection for these cells (Fig. 4*C*), which consist of several populations of CD4^+^ and CD8^+^ T cells, B cells, NK cells, malignant tumor cells, among others. We explored whether BABEL could accurately predict cell type specific expression for marker genes corresponding to each cell type. *CD3E* is a marker gene for T cells, and we see that BABEL recovers this relationship (Fig. 4 *D*, *Left*), much as gene activity scores do (Fig. 4 *D*, *Right*). *CD86*, a marker for myeloid cells, exhibits high specificity and concordance with gene activity scores as well (Fig. 4*E*). BABEL’s predicted single-cell expression of *KLRC1* (killer cell lectin-like receptor C1), a marker for NK cells, is also highly concordant with both annotated cell types and gene activity scores (Fig. 4*F*). In fact, the distribution of BABEL’s imputed *KLRC1* expression appears to be even more specific to the NK cell clusters than gene activity scores—Cicero also predicts *KLRC1* activity in epigenetically similar cytotoxic CD8^+^ T cell clusters (43, 44). We observe similar specificity improvements for *IFNG*, which encodes the interferon gamma protein and is specifically expressed in CD8^+^ activated T cells in scRNA-seq experiments measuring these BCC samples (42). BABEL’s predicted *IFNG* expression is much more localized than the corresponding gene activity scores (Fig. 4*G*) and better approximates empirical scRNA-seq. These two examples highlight concrete cases where BABEL offers clear improvements over traditional gene activity scores. However, BABEL is less accurate if the cell type corresponding to a marker gene is not present in its training data. For example, *KRT14* is a prominent marker for malignant BCC cells, but since there are no basal epithelial cells in BABEL’s training data, BABEL predicts a weak signal for *KRT14* (Fig. 4*H*).

BABEL can also help develop more nuanced understandings for certain cell types in this study. The original scATAC-seq BCC study identified a set of T follicular helper (Tfh) cells that exhibited striking epigenomic similarity to exhausted CD8^+^ T cells (TEx), suggesting that Tfh and TEx differentiation may be driven by a shared regulatory program (10). Using BABEL’s predictions, we find that this Tfh cell cluster may also overexpress common immunosuppressive genes such as *CTLA4* (Fig. 4*I*), *PAK2*, and *FAS* (45–47). While *CTLA4* is also overexpressed in Tfh based on gene activity scores, *PAK2* and *FAS* gene activity scores show weak to no overexpression, despite all three of these genes being overexpressed in Tfh cells in the tissue-matched scRNA-seq study (*SI Appendix*, Fig. S8). Here, BABEL’s ATAC-based inferences help recover an additional parallel between TEx and Tfh cells that is confirmed by scRNA-seq, whereby these cell types not only share epigenetic similarity, but are also similar in their overexpression of immunosuppressive factors.

### BABEL Can Be Extended with Additional Data Modalities.

We finally demonstrate that BABEL can be easily extended to predict additional data modalities, such as protein epitope profiles. We trained an auxiliary protein epitope decoder network using 33,287 jointly profiled RNA and protein epitope measurements of human bone marrow cells, profiled using CITE-seq (23). This protein decoder network leverages BABEL’s pretrained latent representation to infer protein epitope profiles (see *Methods* for more details). The resulting protein decoder network can accurately impute epitope profiles from expression profiles on the test cluster and on novel PBMC cells (*SI Appendix*, Fig. S9 *A* and *B*). As a proof-of-concept, we show that BABEL’s interoperable structure also enables us to generate epitope profiles of a cell from its scATAC-seq profile (*SI Appendix*, Fig. S9 *C*–*E*). BABEL’s ability to make these predictions without explicitly training the ATAC-protein modality pair further illustrates the potential power and versatility of its computational cross-modality translation approach. As more single-cell modalities become available, BABEL can flexibly incorporate these data and link increasingly diverse layers of molecular information, even across modalities yet to be jointly profiled.

## Discussion

BABEL learns a set of neural networks that project single-cell multiomic modalities into a single shared latent representation capturing cellular state, and subsequently uses that latent representation to infer observable genome-wide phenotypes. To achieve our specific task of translating between RNA gene expression and ATAC chromatin accessibility profiles, we leverage best practices for modeling scRNA-seq described in previous works, while introducing two techniques. First, BABEL’s networks for chromatin accessibility data are designed to focus on more biologically relevant intrachromosomal patterns. Second, BABEL’s interoperable encoder/decoder modules effectively leverage paired measurements to learn a meaningful shared latent representation without the use of additional manifold alignment methods. We demonstrate that our resultant BABEL model performs well across a variety of contexts, including held-out test clusters, data generated from different experimental protocols, and even aberrant patient carcinoma samples involving different tissues from those used to train BABEL. Although our evaluation focuses on predicting gene expression from accessibility due to the relative interpretability of gene expression, we also demonstrate the utility of the opposite expression-to-accessibility translation. By providing paired single-cell RNA gene expression and ATAC chromatin accessibility measurements without costly experiments, BABEL can be a valuable tool for hypothesis generation and exploration.

We also carefully investigated potential limitations of BABEL. Across our experiments, BABEL performs best when asked to make predictions on cells for which it has seen similar training examples. When faced with completely foreign cell types such as malignant BCC cells, BABEL does not consistently recover expression of well-known markers like *KRT14*, whereas Cicero’s more conventional gene activity scores do. This limitation is shared by most machine learning approaches; samples deviating too far from the training set often exhibit poor predictive performance (48). As a practical metric, we suggest that aggregate pseudobulk correlation can be a litmus test indicating the quality of BABEL’s imputations on new data. Researchers using BABEL could easily compute such pseudobulk correlations against a growing library of publicly available (bulk or single cell) datasets to evaluate whether BABEL produces trustworthy inferences for their specific experiments. BABEL’s predictions are also limited by the mutual information shared by accessibility and transcriptomic measurements. For example, changes in chromatin accessibility do not always directly or immediately correlate with transcriptomic changes (34); similar variability in BABEL’s predictions is expected.

Beyond translating between expression and chromatin accessibility profiles, we also demonstrated that BABEL provides a computational framework that can be extended to translate between other single-cell modalities via additional encoder and decoder networks. Given additional joint single-cell profiling data, BABEL can act as a pretrained network for transfer learning, especially since its components are interoperable. This can reduce the amount of new data required. New modalities could learn to project into or predict from the same predefined latent space, which simplifies the learning problem and enables translation between unmeasured domain pairs, as shown with BABEL’s ATAC to protein predictions.

BABEL can be combined with other analysis workflows to improve multiomic integration. For example, single-cell gene integration algorithms commonly rely on gene activity scores to bridge the gap between ATAC and RNA modalities, thus enabling cluster-to-cluster or cell-to-cell mapping; substituting gene activities with BABEL’s more accurate expression inferences could greatly improve these integration techniques. BABEL could also be extended by combining its multiomic approach with existing single-omic methods that use variational inference to perform clustering, batch correction, or similar tasks, thus enabling a new class of multimodal algorithms. BABEL’s imputations can expand the representational space of each cell, for example by generating transcriptomic profiles that more clearly illustrate the effects of subtle changes in chromatin accessibility (e.g., *KLRC1* in BCC NK cells). This richer representation might be combined with multiview clustering to more robustly identify groups of related cells.

More broadly, we envision BABEL as a powerful tool for single-cell regulome analysis in an increasingly multiomic world. As methods for measuring different modalities of information within a cell become more available, we will increasingly face trade-offs between the number of modalities, depth of analysis, sample number, and cost. Approaches like BABEL can greatly improve data efficiency beyond the Pareto frontier defined by technological limitations of comeasurement. Once a class of samples has been jointly profiled by single-cell multiomic approaches, scientists can study future instances of such samples (in detailed time courses, perturbation, etc.) with the most economical or technically feasible modality, and infer the remaining information using BABEL. These considerations may be particularly valuable for human clinical samples, which are limited in quantity and perhaps stored in archival formats that do not permit measurement of all modalities (30). By analogy, in 1804, Lewis and Clark took 2 y to explore, map, and journey from St. Louis to the Pacific coast, but travelers today can easily navigate this route in a matter of days, leveraging existing information rather than painstakingly remapping the terrain. We hope that BABEL, along with other multiomic data inference tools, may provide similarly rapid and cost-effective data navigation and insights going forward.

## Materials and Methods

### Data Preprocessing.

We treat single-cell expression data as continuous values. To preprocess expression data, we start with a matrix of unnormalized counts per gene per cell generated using Hg38 (or mm10 for mouse data). These can be produced using tools like CellRanger. We remove genes encoded on sex chromosomes and cells expressing fewer than 200 genes or more than 7,000 genes (2,500 for mouse data). For the combined DM, HSR, and PBMC human data, this retains 34,861/36,417 cells. This retains 34,180/34,774 cells for SHAREseq mouse data, and 10,302/10,309 cells for SNAREseq mouse data. We then size-normalize the data, such that each cell’s counts sum to the median counts per cell. We log-transform the size-normalized counts and standardize these to zero mean and unit variance. We also clip values within the top and bottom 0.5% of the overall distribution. When preprocessing external single-cell expression data for model evaluation, we perform this same series of preprocessing steps.

We treat scATAC-seq data as a binary signal, as we found that a continuous representation made the prediction problem significantly more difficult without providing a meaningful measure of increased accessibility. To preprocess scATAC-seq data, we start with a matrix of cells by peaks, also generated using Hg38 (or mm10 for mouse data). Such matrices can be produced by tools like ArchR or Signac. We remove peaks on sex chromosomes, merge overlapping peaks, and binarize the data by replacing all nonzero values with a value of 1. We then remove peaks occurring in fewer than five cells or more than 10% of cells. This retains 223,897/245,139 peaks for human data, 215,083/238,720 peaks for SNARE-seq, and 326,037/338,304 peaks for SHARE-seq. Removing overly rare peaks helps prevent the model from overfitting on just a handful of examples, while removing overly common peaks helps the model focus on learning important variation between cells. Overall, BABEL is given a filtered, binarized view of the original ATAC peaks.

When predicting on ATAC inputs that may not match the specific peaks BABEL is trained on, we repool the input peaks to match BABEL’s peaks. First, we use liftOver (49) to convert Hg19 coordinates to Hg38 coordinates, if necessary. We then take each input peak, determine which BABEL peak(s) it overlaps, and transfer the source peaks’ values to the overlapped peaks(s) using an “or” operator to combine multiple values. If an input peak has no overlap, it is dropped. For example, if one of BABEL’s ATAC peaks was chr1:1000–2000, and it was given an input with peaks chr1:950–1150 and chr1:1190–2090, the input to BABEL’s chr:1000–2000 peak would be the result of an “or” operator on the values at the two input peaks. This process reduces the resolution of input datasets somewhat, but we find that BABEL is able to make robust predictions regardless.

Many of the described preprocessing steps are done via the Python packages Scanpy, version 1.4.3, and AnnData, version 0.6.22 (50).

### Data Splits.

Training, validation, and test splits were defined by clustering the log-normalized, size-normalized RNA expression data using the Leiden algorithm (51) with a resolution of 1.5. The two largest clusters form validation and test clusters, with remaining cells comprising the training set. Cluster-based data splits reduce the model’s propensity to perform well on validation or test sets by simply “memorizing” a similar cell seen during training. Performance on the test cluster is thus a stronger indicator for how well the model will generalize.

For cross-validation, we evaluate five different combinations of validation and test clusters; no cluster is used more than once as a validation set or as a test set. These folds are created by rotating through the five largest clusters. The first cross-validation split corresponds to using the aforementioned two largest clusters as validation and test and is the model we use when reporting non–cross-validation results in our manuscript.

### BABEL Architecture.

BABEL was implemented using the PyTorch (version 1.2.0) and Skorch (version 0.7.0) Python libraries. BABEL consists of four primary components: two encoder networks, and two decoder networks. Each encoder is responsible for projecting either RNA or ATAC input into the shared latent space, and each of the two decoders infers RNA or ATAC outputs from this shared latent space. These networks are designed to be interoperable—the RNA encoder is compatible with both the RNA decoder and the ATAC decoder, and similarly for the ATAC encoder. Each decoder is also interoperable with both encoders. BABEL does not leverage cluster information in its modeling approach.

The RNA decoder outputs two parameters for each gene, mean and dispersion, which jointly describe the likelihood of each gene’s observed expression under a negative binomial distribution. Coupled with a negative binomial loss, this helps BABEL learn to estimate the true, “de-noised” expression values (the mean parameter) rather than the noisy observed values. The ATAC output consists of a single value per peak bounded between [0, 1]. These ATAC outputs can be binarized using an approach described in SCALE (14), where each entry in the predicted cell by peak matrix is set to “1” if its value is greater than both the corresponding column and row means and “0” otherwise.

For the RNA encoder, we project the input vector of genome-wide expression (*n* = 34,861 for the human model, *n* = 22,541 for SNARE-seq, and *n* = 22,315 for SHARE-seq) to 64 dimensions, followed by a parametric ReLU (PReLU) nonlinear activation. We then project to the 16-dimensional shared latent space, again with PReLU activation. The RNA decoder “inverts” this network architecture. It starts by applying a fully connected layer and PReLU activation to project the 16-dimensional latent representation to 64 dimensions. It then projects this 64-dimensional layer into two outputs matching the input expression vector in size. These are passed through an exponential and softplus activation to estimate the mean and dispersion.

For the ATAC encoder and decoder, we leverage the insight that most DNA accessibility interactions occur on an intrachromosomal level, rather than across different chromosomes (31). Instead of using fully connected layers simultaneously spanning all ATAC peaks across the genome, we use a series of much smaller fully connected layers, each processing only a single chromosome’s peaks. Each chromosome’s peaks are first transformed into a 32-dimensional representation (with PReLU activation), followed by a 16-dimensional representation (with PReLU activation), which is concatenated across all chromosomes. For the human genome with *C* = 22 autosomes, this concatenated layer has 16 × 22 = 352 dimensions. We project this concatenated layer to our 16-dimensional shared latent representation, using a fully connected layer with PReLU activation. This final layer is not chromosome-specific and allows the model to learn some interchromosomal interactions. The ATAC decoder “inverts” the ATAC encoder. It starts by projecting the 16-dimensional latent representation to a *C* × 16 hidden layer (with PReLU), which splits into *C* blocks of 16 dimensions. Each of these *C* blocks passes through an independent set of fully connected layers, mapping to a hidden layer of 32 dimensions with PReLU and finally to the peak predictions with a sigmoid activation. A naive approach with fully genome-wide connections would necessitate nearly *C* times as many parameters (*SI Appendix*, *Supplementary Note*).

### BABEL Training.

The RNA decoder outputs a mean $\hat{y}$ and dispersion θ vector for each gene. These two vectors parameterize the likelihood of observing the measured expression y under a NB distribution, as shown below (Γ denotes the gamma function):

$$P\left(y;\hat{y},\theta \right)=\frac{\text{Γ}\left(y+\theta \right)}{y!\text{Γ}\left(y\right)}{\left(\frac{\theta }{\theta +\hat{y}}\right)}^{\theta }{\left(\frac{\hat{y}}{\theta +\;\hat{y}}\right)}^{y}.$$

Intuitively, we want to find the mean and dispersion $\hat{y},\theta$ that maximize the likelihood of the observed data. This is equivalent to minimizing the negative log likelihood, which we use as our loss as follows (ϵ is a small constant for numerical stability):

$$\begin{array}{c} L_{NB}\left(y;\hat{y},\theta \right)=-\theta \left(\log\left(\theta +\epsilon \right)-\log\left(\theta +\;\hat{y}\right)\right)-y\left(\log\left(\hat{y}+\epsilon \right)-\text{log}\left(\theta +\hat{y}\right)\right)\\ -\text{log}\;\Gamma \left(y+\theta \right)+\log\text{Γ}\left(y+1\right)+\log\text{Γ}\left(\theta +\epsilon \right). \end{array}$$

Since the ATAC decoder generates a binary prediction for each peak across the genome, we use a BCE loss where x represents the measured ATAC signal at each bin, and $\hat{x}$ represents our model’s prediction:

$$L_{BCE}\left(x;\hat{x}\right)=-\left(x\log\hat{x}+\left(1-x\right)\log\left(1-\hat{x}\right)\right).$$

Given these building blocks, we can formulate the overall loss function for our model. Recall that we want each encoder to be composable with either decoder, such that all our networks are interoperable. We express this in our overall loss L by including a term for each of our four encoder/decoder combinations. Here, r and a denote the measured RNA and ATAC signals, respectively; subscripts denote the source modality used to infer either RNA or ATAC (e.g., $r_{ATAC}$ represents the inferred RNA values from ATAC input):

$$L=L_{NB}\left(r,\;r_{RNA}\right)+\beta L_{BCE}\left(a,\;a_{ATAC}\right)+\beta L_{BCE}\left(a,\;a_{RNA}\right)+L_{NB}\left(r,\;r_{ATAC}\right).$$

The first two terms encapsulate how well our model can “reconstruct” the RNA and ATAC inputs via intradomain inference. The latter two terms encapsulate how well our model performs cross-domain prediction (i.e., inferring ATAC profiles from RNA expression and vice versa). The constant β ensures that the BCE and NB losses are numerically within the same order of magnitude. Based on manually examining the magnitude of loss components over the first few training epochs, we set β=1.33 for all results described in this paper.

We trained our model using the Adam optimizer (52) with a batch size of 512 and a learning rate of 0.01. We reduce the learning rate and perform early stopping based on validation set loss. We also use batch normalization and gradient clipping to aid in training stability.

### Baseline KNN Model.

We implemented two KNN models to contextualize BABEL’s ATAC to RNA and RNA to ATAC translation performance. Both models compare each query cell to every cell in its training set, computing pairwise Euclidean distance in feature space (binarized counts for ATAC to RNA, log-scaled size-normalized counts for RNA to ATAC). KNN takes the top *k* = 10 “closest” training cells and averages them to generate a prediction. The value *k* = 10 was chosen because it exhibits relatively good performance. Taking *k* = 1, for example, performs substantially worse with a Pearson correlation of 0.12 when inferring ATAC to RNA on the test set, and an AUROC of 0.59 when inferring RNA to ATAC, compared to 0.27 and 0.84 for *k* = 10.

### Evaluation Metrics and Statistical Analysis.

We use several metrics to evaluate BABEL’s outputs. As we binarize ATAC chromatin accessibility, we use AUROC to evaluate the quality of our ATAC predictions. As we consider RNA expression to be continuous, we use Pearson’s and Spearman’s correlations to evaluate the quality of our RNA expression predictions and gene activity scores. Scatterplots and density scatterplots are labeled with Pearson’s correlation values. Since scRNA-seq analyses like clustering and dimensionality reduction are predominantly performed on log-counts, all such correlations are computed and shown in log space. For both correlation and AUROC, we effectively consider each gene/peak in each cell a separate observation/prediction.

To generate pseudobulk RNA expression signatures, we average the size-normalized, log-scaled expression of each gene across all cells. We then compare these per-gene means across similar samples using Pearson’s correlation. In cases where we compare bulk expression across two different genome assemblies (i.e., Hg19 and Hg38), the pseudobulk is reported on the intersection of genes in the assemblies.

All metrics are calculated using the Python packages Sklearn version 0.21.2 (53), SciPy version 1.2.1 (54), and NumPy. When performing UMAP dimension reduction on single-cell expression data, we use the size-normalized, log-scaled expression. UMAP was calculated using Scanpy (50) using hyperparameters taken from Seurat’s default settings. When visualizing single-cell ATAC-seq measurements, we use a term-frequency times inverse document-frequency (F-IDF) transform before applying UMAP.

### Analysis on Jointly Profiled Multiomic Datasets.

Single-cell paired RNA and ATAC-seq libraries for GM12878 were generated on the 10x Chromium Single-Cell Multiome ATAC + Gene Expression platform following the manufacturer’s protocol and sequenced on an Illumina NovaSeq 6000. The single-cell paired RNA and ATAC-seq reads were aligned to the hg38 reference genome using cellranger-arc count (10x Genomics, version 1.0.0).

We compare BABEL’s ATAC to RNA predictions to gene activity scores generated by ArchR version 0.9.5 (24) and MAESTRO version 1.2.1 (25). We used ArchR’s default parameters with the Hg38 assembly and MAESTRO’s “enhanced” estimation mode with default parameters and the Hg38 assembly. We also compare BABEL’s RNA to ATAC predictions to similar outputs generated by BIRD (32, 33). We use BIRD v1.1.1 and the authors’ preconfigured human Hg38 v1.4 model.

### Unpaired PBMC Analysis.

Single-cell ATAC-seq healthy PBMC data for analyzing BABEL’s ability to infer single-cell expression profiles from scATAC-seq data are available from 10x Genomics. We used the filtered peak matrix, passed through liftOver for coordinate conversion from Hg19 to Hg38, as input to BABEL. Cell type annotations were obtained using Signac, version 1.0.0, for scATAC-seq analysis. UMAP visualization is generated from BABEL’s imputed RNA expression signatures after size-normalization and log transformation. To contextualize the reconstructed gene expression results, we used healthy PBMC scRNA-seq data publicly available through 10x Genomics, which was then analyzed and visualized using Seurat v3.2.0.

Single-cell RNA-seq PBMC data for analyzing BABEL’s ability to infer scATAC-seq accessibility profiles from transcriptomic data are available from 10x Genomics. Cluster assignments were obtained via Seurat. BABEL was used to infer genome-wide chromatin accessibility profiles. We then used ScanPy to identify the top 10 marker peaks for each cluster. Specifically, this trains a logistic regression model for each cluster, attempting to classify cells as “in-cluster” versus “not-in-cluster.” This approach is powerful for scRNA-seq data (55) and is also showcased in the Signac PBMC scATAC-seq vignette. The peaks with the largest weights in the logistic regression model are regarded as more informative. These peaks were associated with their nearest annotated transcript within 10 kilobases using Ensembl’s v100 annotation (56).

### Basal Cell Carcinoma Analysis.

When evaluating BABEL’s ATAC to RNA performance on BCC samples, we use scATAC-seq data as published in the original manuscript (10), passed through liftOver for Hg38 conversion. UMAP plots for this dataset are based on the scATAC-seq measurements. Gene expression is predicted by BABEL based on single-cell ATAC and is size-normalized and log-transformed prior to analysis and plotting.

To find overexpressed marker genes corresponding to a cluster of cells, we use Scanpy to perform a Wilcoxon rank-sum test with Benjamini–Hochberg correction, comparing the (predicted) expression of each gene within the cluster against its expression in all other cells. We then take the top 100 most significant results (or fewer if doing so would include nonsignificant results with adjusted *P* > 0.05). The resulting list can then be manually examined for overexpression of key genes, which we explore and report in our manuscript.

### Extending BABEL to Predict Protein Epitopes.

To preprocess single-cell epitope data, we apply a centered log-ratio (CLR) transformation to each cell’s protein counts. CLR normalization is commonly used for modeling epitope data (5, 57). The CLR transformation for a count vector x (with added pseudocounts) measuring n proteins in a cell is given by the following expression, where g(x) denotes the geometric mean:

$$CLR\left(x\right)=\ln\left[\frac{x_{1}}{g\left(x\right)},\;\ldots ,\frac{x_{n}}{g\left(x\right)}\right].$$

Additionally, we filter cells based on their expression profiles using the preprocessing cutoffs discussed in the data preprocessing section above.

To extend BABEL to predict CLR-normalized protein epitopes, an auxiliary protein decoder network takes, as input, BABEL’s 16-dimensional latent representation, and predicts CLR-normalized protein counts. This decoder network consists of fully connected layers projecting from hidden layers of size 16, 64, to an output of 25 dimensions, with hyperbolic tangent (tanh) activations save for the final output layer, which uses an identity activation. This protein decoder network is trained on CITE-seq paired single-cell protein epitope and RNA expression measurements using a mean-squared-error loss. In this process, BABEL’s encoder networks and latent representation is fixed. Training/validation/test data splits are defined by single-cell expression clusters, as was done to train the main BABEL model. To generate protein epitope predictions from scATAC-seq data, we first use BABEL to infer a corresponding expression profile, and then infer single-cell epitopes from this inferred expression profile.

### Plotting.

All plots were generated using Matplotlib (58), Seaborn (https://seaborn.pydata.org) adjustText (https://github.com/Phlya/adjustText), mpl-scatter-density (https://github.com/astrofrog/mpl-scatter-density), Astropy (59, 60), and Scanpy (50) libraries under Python 3.7.

## Supplementary Material

Supplementary File

## Data Availability

Human data jointly profiling PBMC cells’ expression and chromatin accessibility is available from 10x Genomics’ data portal (https://support.10xgenomics.com/single-cell-multiome-atac-gex/datasets/1.0.0/pbmc_granulocyte_sorted_10k). Human data jointly profiling DM and HSR cells’ expression and chromatin accessibility is available through Gene Expression Omnibus (GEO) (accession no. GSE160148). Human data jointly profiling GM12878 cells is available through GEO (accession no. GSE166797). SNARE-seq mouse data are publicly available at GEO (accession no. GSE126074). SHARE-seq mouse data are available at GEO (accession no. GSE140203). PBMC scATAC-seq data are available through 10x Genomics’ data portal (https://support.10xgenomics.com/single-cell-atac/datasets/1.2.0/atac_v1_pbmc_10k). A tissue-matched 3k PBMC scRNA-seq dataset is available through 10x Genomics’ data portal (https://support.10xgenomics.com/single-cell-multiome-atac-gex/datasets/1.0.0/pbmc_granulocyte_sorted_10k). PBMC scRNA-seq data for evaluating RNA to ATAC translation is available through 10x Genomics’ data portal (https://support.10xgenomics.com/single-cell-gene-expression/datasets/3.0.0/pbmc_10k_v3). BCC scATAC-seq data are available through GEO (accession no. GSE129785), with Cicero gene activity scores available on the authors’ GitHub. Corresponding BCC scRNA-seq data are available through GEO (accession no. GSE123813). GM12878 scRNA-seq data are available from GEO (accession no. GSE126321). CITE-seq data on human bone marrow cells is available through GEO (accession no. GSE128639). CITE-seq data on PBMCs is available through 10x Genomics’ data portal (https://support.10xgenomics.com/single-cell-gene-expression/datasets/3.1.0/5k_pbmc_protein_v3_nextgem). All study data are included in the article and/or supporting information. All code required to reproduce the BABEL model and our reported results, including data preprocessing and model training, have been deposited on GitHub (https://github.com/wukevin/babel).

## References

[1] T. Stuart, R. Satija, Integrative single-cell analysis. Nat. Rev. Genet. 20, 257–272 (2019). 30696980 10.1038/s41576-019-0093-7

[2] A. Ma, A. McDermaid, J. Xu, Y. Chang, Q. Ma, Integrative methods and practical challenges for single-cell multi-omics. Trends Biotechnol. 38, 1007–1022 (2020). 32818441 10.1016/j.tibtech.2020.02.013 PMC7442857

[3] S. Chen, B. B. Lake, K. Zhang, High-throughput sequencing of the transcriptome and chromatin accessibility in the same cell. Nat. Biotechnol. 37, 1452–1457 (2019). 31611697 10.1038/s41587-019-0290-0 PMC6893138

[4] J. Cao., Joint profiling of chromatin accessibility and gene expression in thousands of single cells. Science 361, 1380–1385 (2018). 30166440 10.1126/science.aau0730 PMC6571013

[5] M. Stoeckius., Simultaneous epitope and transcriptome measurement in single cells. Nat. Methods 14, 865–868 (2017). 28759029 10.1038/nmeth.4380 PMC5669064

[6] X. Chen., Joint single-cell DNA accessibility and protein epitope profiling reveals environmental regulation of epigenomic heterogeneity. Nat. Commun. 9, 4590 (2018). 30389926 10.1038/s41467-018-07115-y PMC6214962

[7] E. P. Mimitou., Scalable, multimodal profiling of chromatin accessibility and protein levels in single cells. bioRxiv [Preprint] (2020). 10.1073/pnas.2023070118 (Accessed 9 October 2020). PMC8763625 34083792

[8] J. Lee, D. Y. Hyeon, D. Hwang, Single-cell multiomics: Technologies and data analysis methods. Exp. Mol. Med. 52, 1428–1442 (2020). 32929225 10.1038/s12276-020-0420-2 PMC8080692

[9] D. Lähnemann., Eleven grand challenges in single-cell data science. Genome Biol. 21, 31 (2020). 32033589 10.1186/s13059-020-1926-6 PMC7007675

[10] A. T. Satpathy., Massively parallel single-cell chromatin landscapes of human immune cell development and intratumoral T cell exhaustion. Nat. Biotechnol. 37, 925–936 (2019). 31375813 10.1038/s41587-019-0206-z PMC7299161

[11] G. Eraslan, L. M. Simon, M. Mircea, N. S. Mueller, F. J. Theis, Single-cell RNA-seq denoising using a deep count autoencoder. Nat. Commun. 10, 390 (2019). 30674886 10.1038/s41467-018-07931-2 PMC6344535

[12] M. Amodio., Exploring single-cell data with deep multitasking neural networks. Nat. Methods 16, 1139–1145 (2019). 31591579 10.1038/s41592-019-0576-7 PMC10164410

[13] R. Lopez, J. Regier, M. B. Cole, M. I. Jordan, N. Yosef, Deep generative modeling for single-cell transcriptomics. Nat. Methods 15, 1053–1058 (2018). 30504886 10.1038/s41592-018-0229-2 PMC6289068

[14] L. Xiong., SCALE method for single-cell ATAC-seq analysis via latent feature extraction. Nat. Commun. 10, 4576 (2019). 31594952 10.1038/s41467-019-12630-7 PMC6783552

[15] M. Amodio, S. Krishnaswamy, Magan: Aligning biological manifolds. Proc. Mach. Learn. Res. 80, 215–223 (2018).

[16] J. Liu, Y. Huang, R. Singh, J.-P. Vert, W. S. Noble, “Jointly embedding multiple single-cell omics measurements” in 19th International Workshop on Algorithms in Bioinformatics (WABI2019), K. T. Huber, D. Gusfield, Eds. (Leibniz International Proceedings in Informatics, 2019), 143, p. 10:1-10:13. 10.4230/LIPIcs.WABI.2019.10 PMC8496402 34632462

[17] K. D. Yang, C. Uhler, Multi-domain translation by learning uncoupled autoencoders. arXiv [Preprint] (2019). https://arxiv.org/abs/1902.03515 (Accessed 28 September 2020).

[18] K. D. Yang., Multi-domain translation between single-cell imaging and sequencing data using autoencoders. Nat. Commun. 12, 31 (2021). 33397893 10.1038/s41467-020-20249-2 PMC7782789

[19] L. Zhang., Deep learning-based multi-omics data integration reveals two prognostic subtypes in high-risk neuroblastoma. Front. Genet. 9, 477 (2018). 30405689 10.3389/fgene.2018.00477 PMC6201709

[20] A. Gayoso., Joint probabilistic modeling of paired transcriptome and proteome measurements in single cells. bioRxiv [Preprint] (2020). 10.1101/2020.05.08.083337 (Accessed 12 December 2020).

[21] R. Gala., “A coupled autoencoder approach for multi-modal analysis of cell types” in Advances in Neural Information Processing Systems 32, H. Wallach, Ed. . (NeurIPS Foundation, 2019), pp. 9267–9276.

[22] R. Shen, A. B. Olshen, M. Ladanyi, Integrative clustering of multiple genomic data types using a joint latent variable model with application to breast and lung cancer subtype analysis. Bioinformatics 25, 2906–2912 (2009). 19759197 10.1093/bioinformatics/btp543 PMC2800366

[23] T. Stuart., Comprehensive integration of single-cell data. Cell 177, 1888–1902.e21 (2019). 31178118 10.1016/j.cell.2019.05.031 PMC6687398

[24] J. M. Granja., Archr: An integrative and scalable software package for single-cell chromatin accessibility analysis. bioRxiv:2020.04.28.066498 (2020).

[25] C. Wang., Integrative analyses of single-cell transcriptome and regulome using MAESTRO. Genome Biol. 21, 198 (2020). 32767996 10.1186/s13059-020-02116-x PMC7412809

[26] J. D. Welch, A. J. Hartemink, J. F. Prins, MATCHER: Manifold alignment reveals correspondence between single cell transcriptome and epigenome dynamics. Genome Biol. 18, 138 (2017). 28738873 10.1186/s13059-017-1269-0 PMC5525279

[27] Z. Duren., Integrative analysis of single-cell genomics data by coupled nonnegative matrix factorizations. Proc. Natl. Acad. Sci. U.S.A. 115, 7723–7728 (2018). 29987051 10.1073/pnas.1805681115 PMC6065048

[28] G. L. Stein-O’Brien., Enter the matrix: Factorization uncovers knowledge from omics. Trends Genet. 34, 790–805 (2018). 30143323 10.1016/j.tig.2018.07.003 PMC6309559

[29] S. Jin, L. Zhang, Q. Nie, scAI: An unsupervised approach for the integrative analysis of parallel single-cell transcriptomic and epigenomic profiles. Genome Biol. 21, 25 (2020). 32014031 10.1186/s13059-020-1932-8 PMC6996200

[30] N. E. Navin, The first five years of single-cell cancer genomics and beyond. Genome Res. 25, 1499–1507 (2015). 26430160 10.1101/gr.191098.115 PMC4579335

[31] J. Dekker, T. Misteli, Long-range chromatin interactions. Cold Spring Harb. Perspect. Biol. 7, a019356 (2015). 26430217 10.1101/cshperspect.a019356 PMC4588061

[32] W. Zhou., Genome-wide prediction of DNase I hypersensitivity using gene expression. Nat. Commun. 8, 1038 (2017). 29051481 10.1038/s41467-017-01188-x PMC5715040

[33] W. Zhou, Z. Ji, W. Fang, H. Ji, Global prediction of chromatin accessibility using small-cell-number and single-cell RNA-seq. Nucleic Acids Res. 47, e121 (2019). 31428792 10.1093/nar/gkz716 PMC6821224

[34] S. Ma., Chromatin potential identified by shared single-cell profiling of RNA and chromatin. Cell 183, 1103–1116.e20 (2020). 33098772 10.1016/j.cell.2020.09.056 PMC7669735

[35] E. Becht., Dimensionality reduction for visualizing single-cell data using UMAP. Nat. Biotechnol. 37, 38–44 (2018). 10.1038/nbt.4314 30531897

[36] L. McInnes, J. Healy, N. Saul, L. Großberger, Umap: Uniform manifold approximation and projection. J. Open Source Softw. 3, 861 (2018).

[37] H. Georgiev, I. Ravens, G. Papadogianni, G. Bernhardt, Coming of age: Cd96 emerges as modulator of immune responses. Front. Immunol. 9, 1072 (2018). 29868026 10.3389/fimmu.2018.01072 PMC5966540

[38] F. Liu., CD96, a new immune checkpoint, correlates with immune profile and clinical outcome of glioma. Sci. Rep. 10, 10768 (2020). 32612110 10.1038/s41598-020-66806-z PMC7330044

[39] Y. Li., Pan-cancer characterization of immune-related lncRNAs identifies potential oncogenic biomarkers. Nat. Commun. 11, 1000 (2020). 32081859 10.1038/s41467-020-14802-2 PMC7035327

[40] X. Feng., Foxp1 is an essential transcriptional regulator for the generation of quiescent naive T cells during thymocyte development. Blood 115, 510–518 (2010). 19965654 10.1182/blood-2009-07-232694 PMC2810984

[41] H. A. Pliner., Cicero predicts *cis*-regulatory DNA interactions from single-cell chromatin accessibility data. Mol. Cell 71, 858–871.e8 (2018). 30078726 10.1016/j.molcel.2018.06.044 PMC6582963

[42] K. E. Yost., Clonal replacement of tumor-specific T cells following PD-1 blockade. Nat. Med. 25, 1251–1259 (2019). 31359002 10.1038/s41591-019-0522-3 PMC6689255

[43] A. Kurioka, P. Klenerman, C. B. Willberg, Innate-like CD8^+^ T-cells and NK cells: Converging functions and phenotypes. Immunology 154, 547–556 (2018). 10.1111/imm.12925 PMC6050209 29542114

[44] E. Narni-Mancinelli, E. Vivier, Y. M. Kerdiles, The ‘T-cell-ness’ of NK cells: Unexpected similarities between NK cells and T cells. Int. Immunol. 23, 427–431 (2011). 21665959 10.1093/intimm/dxr035

[45] E. I. Buchbinder, A. Desai, CTLA-4 and PD-1 pathways: Similarities, differences, and implications of their inhibition. Am. J. Clin. Oncol. 39, 98–106 (2016). 26558876 10.1097/COC.0000000000000239 PMC4892769

[46] K. L. O’Hagan, S. D. Miller, H. Phee, Pak2 is essential for the function of Foxp3^+^ regulatory T cells through maintaining a suppressive Treg phenotype. Sci. Rep. 7, 17097 (2017). 29213081 10.1038/s41598-017-17078-7 PMC5719048

[47] A. Yamada, R. Arakaki, M. Saito, Y. Kudo, N. Ishimaru, Dual role of Fas/FasL-mediated signal in peripheral immune tolerance. Front. Immunol. 8, 403 (2017). 28424702 10.3389/fimmu.2017.00403 PMC5380675

[48] D. Amodei., Concrete problems in AI safety. arXiv [Preprint] (2016). https://arxiv.org/abs/1606.06565 (Accessed 28 September 2020).

[49] A. S. Hinrichs., The UCSC genome browser database: Update 2006. Nucleic Acids Res. 34, D590–D598 (2006). 16381938 10.1093/nar/gkj144 PMC1347506

[50] F. A. Wolf, P. Angerer, F. J. Theis, SCANPY: Large-scale single-cell gene expression data analysis. Genome Biol. 19, 15 (2018). 29409532 10.1186/s13059-017-1382-0 PMC5802054

[51] V. A. Traag, L. Waltman, N. J. van Eck, From Louvain to Leiden: Guaranteeing well-connected communities. Sci. Rep. 9, 5233 (2019). 30914743 10.1038/s41598-019-41695-z PMC6435756

[52] D. P. Kingma, J. Ba, Adam: A method for stochastic optimization. arXiv [Preprint] (2014). https://arxiv.org/abs/1412.6980 (Accessed 28 September 2020).

[53] F. Pedregosa., Scikit-learn: Machine learning in Python. J. Machine Learn. Res. 12, 2825–2830 (2011).

[54] E. Jones, T. Oliphant, P. Peterson, SciPy: Open source scientific tools for Python. http://www.scipy.org/. Accessed 28 September 2020.

[55] V. Ntranos, L. Yi, P. Melsted, L. Pachter, Identification of transcriptional signatures for cell types from single-cell RNA-seq. bioRxiv [Preprint] (2018). 10.1101/258566 (Accessed 17 December 2020).

[56] F. Cunningham., Ensembl 2019. Nucleic Acids Res. 47, D745–D751 (2019). 30407521 10.1093/nar/gky1113 PMC6323964

[57] Z. Zhou, C. Ye, J. Wang, N. R. Zhang, Surface protein imputation from single cell transcriptomes by deep neural networks. Nat. Commun. 11, 651 (2020). 32005835 10.1038/s41467-020-14391-0 PMC6994606

[58] J. D. Hunter, Matplotlib: A 2D graphics environment. Comput. Sci. Eng. 9, 90–95 (2007).

[59] T. P. Robitaille., Astropy: A community Python package for astronomy. Astron. Astrophys. 558, A33 (2013).

[60] A. M. Price-Whelan., The Astropy Project: Building an open-science project and status of the v2.0 core package. Astron. J. 156, 123 (2018).

