# Supplementary material for: BABEL enables cross-modality translation between multiomic profiles at single-cell resolution
Source: Proc Natl Acad Sci U S A. 2021 Apr 7;118(15):e2023070118. doi: 10.1073/pnas.2023070118 (PMC8054007; doi:10.1073/pnas.2023070118)
Supplement: Supplementary File [file pnas.2023070118.sapp.pdf]

Supplementary Tables and Figures

**Test Cluster**	Metric	16-dim	32-dim	64-dim
RNA > RNA	Pearson's *r*	0.64	0.64	0.64
ATAC > RNA		0.62	0.62	0.62
RNA > RNA	Spearman's *r*	0.35	0.35	0.35
ATAC > RNA		0.35	0.35	0.35
ATAC > ATAC	AUROC	0.94	0.94	0.94
RNA > ATAC		0.92	0.92	0.92

Supplementary Table S1: BABEL performance on test cluster when trained using varying latent dimension sizes. Increasing the size of the latent representation does not measurably improve model performance on the held-out test cluster. Thus, we elect to train BABEL using a 16-dimensional hidden layer to provide the most implicit regularization.

**GM12878**	Metric	BABEL	Independent Models
RNA > RNA	Pearson's *r*	0.59	0.54
ATAC > RNA		0.59	0.59
RNA > RNA	Spearman's *r*	0.37	0.37
ATAC > RNA		0.38	0.38
ATAC > ATAC	AUROC	0.85	0.85
RNA > ATAC		0.65	0.63

Supplementary Table S2: Comparison of BABEL against independent models without shared encoders/decoders on GM12878. The independent models are designed to be architectural copies of the corresponding encoder/decoder combination in BABEL (i.e., the same number of input/output dimensions, number of layers, non-linear activations, etc.), except that the four independent models are completely separate and are only trained to perform a singular task. In contrast, BABEL requires that encoders and decoders are interoperable, in that each decoder can be composed with either encoder to produce a reasonable output. BABEL outperforms the independent models by a moderate margin for both RNA to RNA and RNA to ATAC translation and performs similarly for ATAC to RNA and ATAC to ATAC. The other advantage of BABEL is that due to the interoperability of each subcomponent network, the entire BABEL model contains half the parameters of the four independent models combined. This makes BABEL faster and easier to train. Overall, this suggests that training interoperable network modules can help BABEL to learn a more efficient, generalizable representation.

Test Cluster	Metric	Fold 1	Fold 2	Fold 3	Fold 4	Fold 5
	Train cells	28408	29323	29524	29718	28615
	Valid cells	2881	1979	1966	1778	1772
	Test cells	1979 (PBMC)	1966 (HSR)	1778 (HSR)	1772 (DM)	2881 (HSR)
RNA > RNA	Pearson's *r*	0.64	0.82	0.81	0.84	0.84
ATAC > RNA		0.62	0.80	0.78	0.81	0.82
RNA > RNA	Spearman's *r*	0.35	0.55	0.54	0.58	0.60
ATAC > RNA		0.35	0.55	0.53	0.58	0.59
ATAC > ATAC	AUROC	0.94	0.90	0.90	0.91	0.91
RNA > ATAC		0.92	0.87	0.87	0.88	0.87

Supplementary Table S3: BABEL cross-validation performance across different held-out test clusters. Each fold represents unique validation and test clusters, cycling through the 5 largest clusters in our data. The top three rows describe the size and composition of each cross-validation fold. Performance figures are reported on each fold's test cluster. Intra-domain inference is shown as a sanity check, as it is not the primary focus of our work. We observe consistent performance across all folds 2-5; fold 1 exhibits improved ATAC inference performance, but with reduced RNA inference performance. Such variability is expected, as each fold represents evaluation on an entirely different held-out cell type. Notably, this variability does not extend to evaluating BABEL in other contexts, such as GM12878 (Supplementary Table S4) or unpaired PBMC data (Supplementary Table S5). Fold 1 corresponds to the primary model we use throughout the paper, as it is ordinally first and corresponds to using the first and second largest clusters as validation/test sets, respectively.

**GM12878**	Metric	Fold 1	Fold 2	Fold 3	Fold 4	Fold 5
RNA > RNA	Pearson's *r*	0.59	0.58	0.57	0.59	0.60
ATAC > RNA		0.59	0.57	0.54	0.58	0.58
RNA > RNA	Spearman's r	0.37	0.37	0.38	0.38	0.37
ATAC > RNA		0.38	0.38	0.38	0.38	0.38
ATAC > ATAC	AUROC	0.85	0.85	0.84	0.85	0.84
RNA > ATAC		0.65	0.67	0.67	0.66	0.62

Supplementary Table S4: BABEL performance on the GM12878 paired ATAC/RNA data. These folds correspond to the same data splits used in Supplementary Table S3. The model itself changes as its training and validation sets vary, and each model is evaluated on the same set of 7,361 GM12878 cells. We observe very little variation in performance, which indicates that BABEL is robust to variation in its training set and exhibits similarly strong generalizability regardless.

**Unpaired PBMC performance**	Fold 1	Fold 2	Fold 3	Fold 4	Fold 5
Pseudo-bulk Pearson's *r*	0.89	0.90	0.91	0.91	0.89

Supplementary Table S5: BABEL's pseudo-bulk concordance on unpaired PBMC cells across cross-validation folds. As in Supplementary Tables 3 and 4, each fold represents a different model trained using a different training and validation set spanning DM, HSR, and PBMC cells. We show pseudo-bulk correlation here, which measures the concordance in the average expression per gene across cells, as this dataset is not paired. Specifically, we compare BABEL's pseudo-bulk expression against a reference dataset profiling PBMCs with scRNA-seq, reiterating the comparison drawn in Figure 3. Despite variations in the training set, BABEL exhibits uniformly high performance across all folds. This further indicates that BABEL is a robust model that consistently learns generalizable patterns.

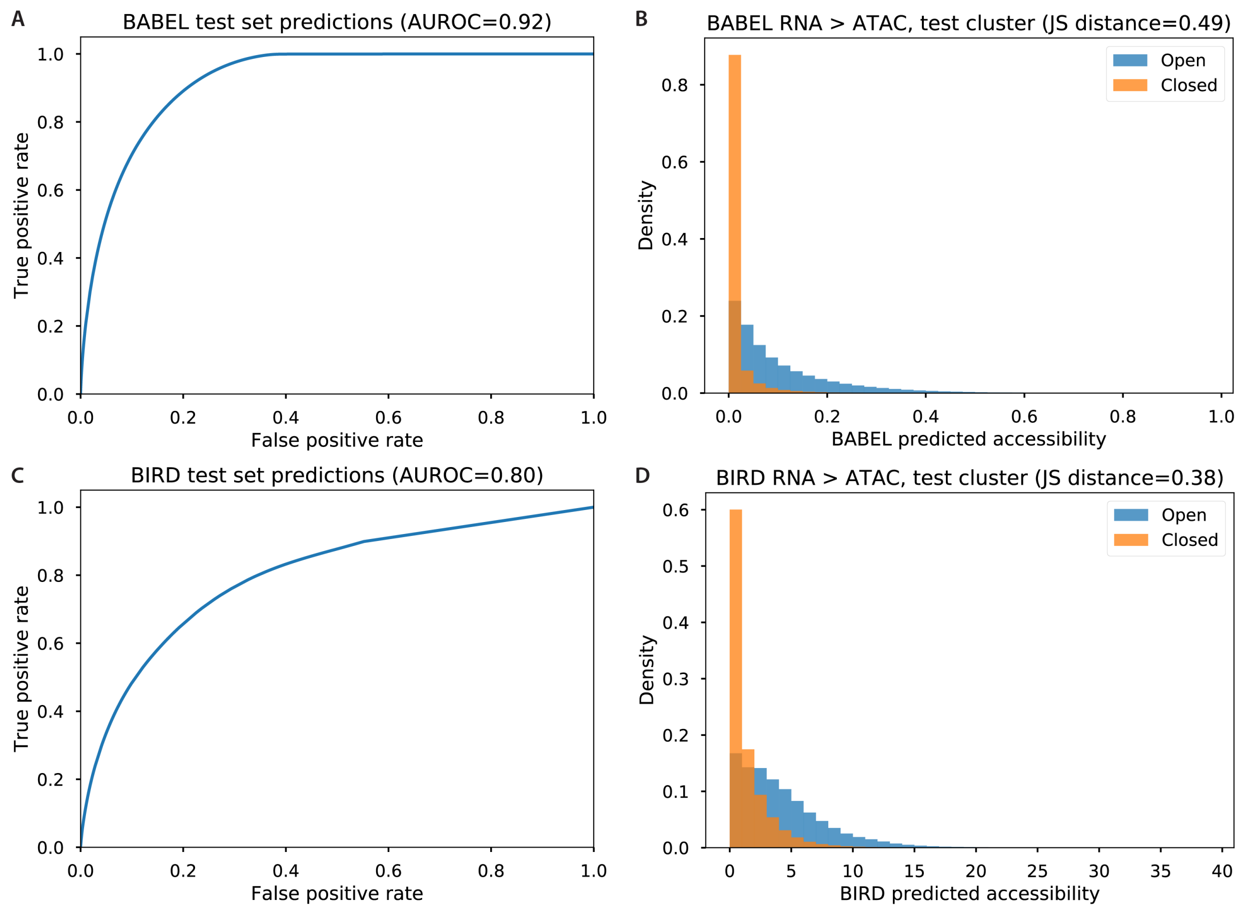

Supplementary Figure S1: BABEL compared to BIRD, evaluated on the held-out test cluster. This cluster consists of 1,979 jointly profiled PBMC cells. (A) BABEL's AUROC and corresponding predicted probabilities (B). (C) BIRD's AUROC and corresponding predicted continuous accessibilities (D). We evaluate all model predictions with respect to binarized ATAC measurements (where nonzero accessibility is universally regarded as 1, and 0 otherwise), considering each peak across each cell in the test cluster. A binarized reference allows us to compute metrics like AUROC (by evaluating the true/false positive rate of various cutoffs) despite BIRD and BABEL having different output domains (i.e., fully continuous vs. probabilities, respectively). Both tools predict greater accessibility for regions with empirically measured open regions. However, BABEL produces greater separation in its predictions corresponding to open and closed regions, as evidenced by its higher AUROC (A, C), as well as a greater Jensen-Shannon (JS) distance separating the distribution of its predictions (B, D, histograms colored by binarized measured accessibility).

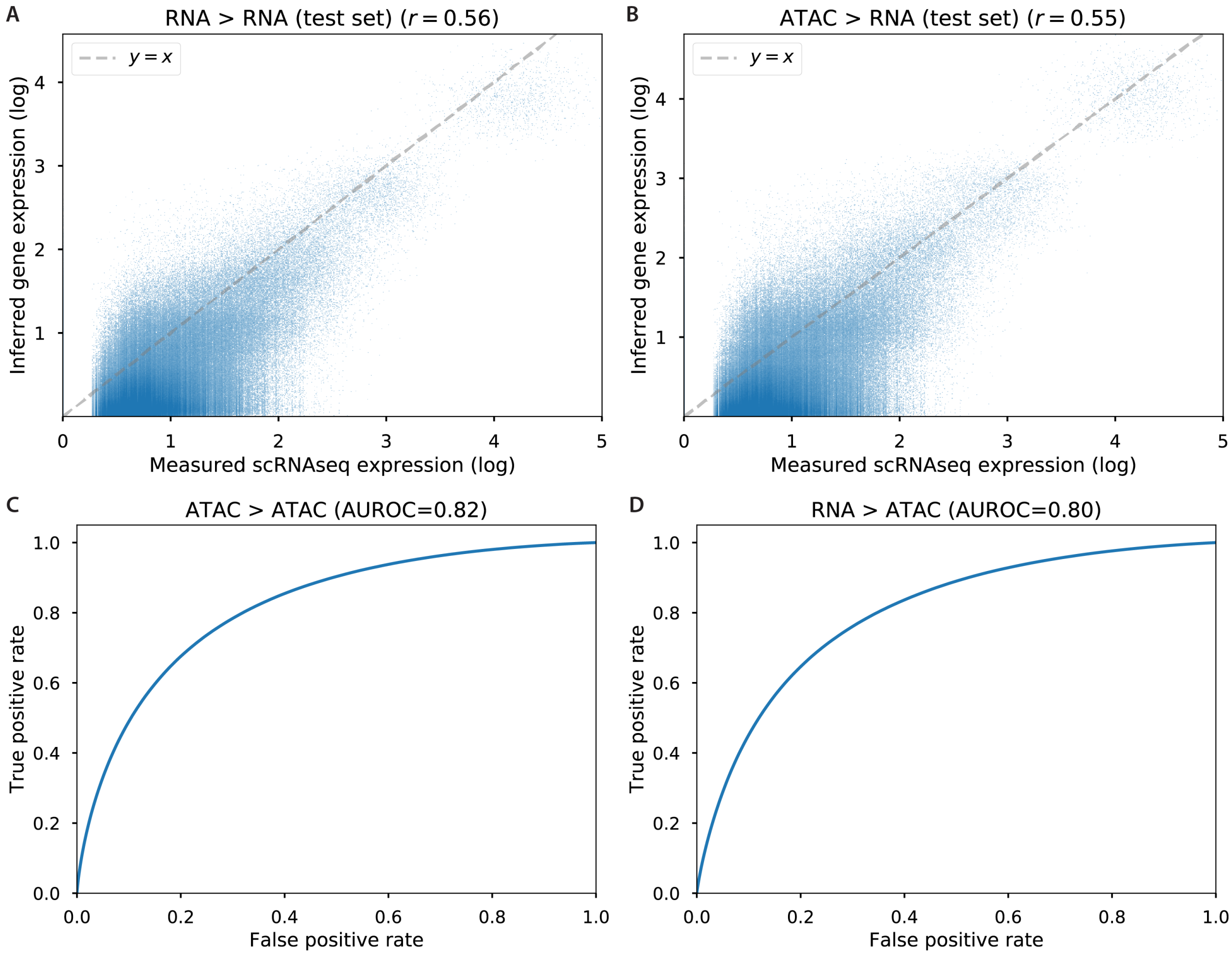

Supplementary Figure S2: BABEL's performance when trained and tested on the SNARE-seq mouse data. Test cluster consists of 1,308 cells. (A) Intra-domain RNA to RNA inference performance. This density scatterplot shows the expression of each gene in each cell within the held-out test cluster; the x-axis represents the empirically measured expression of that gene in that cell, while the y-axis represents the inferred expression. (B) Cross-domain ATAC to RNA inference performance, formatted as in panel (A). (C) ATAC to ATAC intra-domain inference. (D) RNA to ATAC inference performance. We observe that all four possible encoder/decoder combinations perform well, indicating that BABEL can be successfully trained on non-human data generated using different experimental protocols.

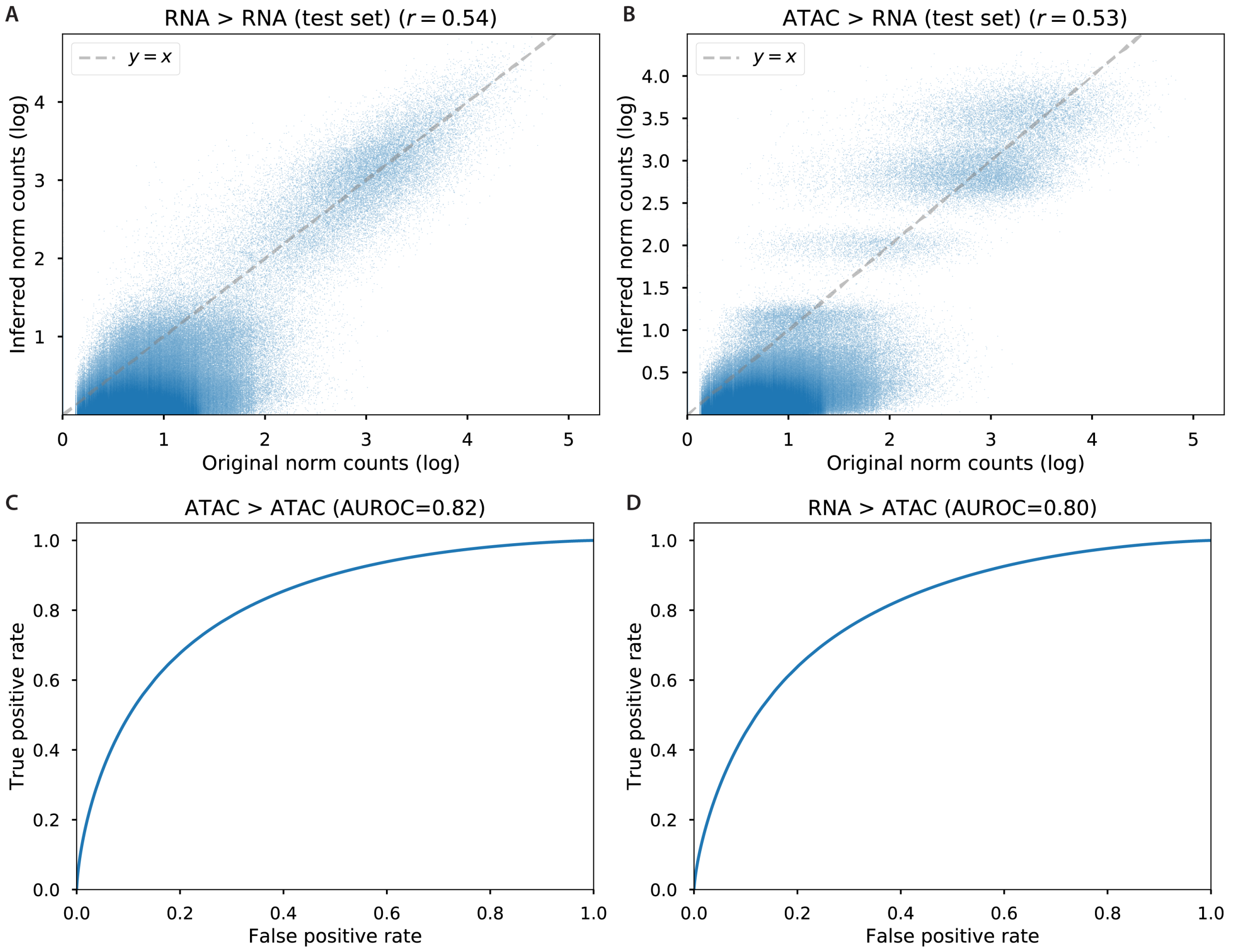

Supplementary Figure S3: BABEL's performance when trained and tested on SHARE-seq mouse data, which jointly profiles 34 thousand murine skin cells. All results shown on the test cluster, which consists of 3,670 cells. (A) Intra-domain RNA to RNA inference performance evaluated for each gene in each cell, with Pearson's correlation. In this density scatterplot, increased color darkness indicates more points at that location. The x-axis represents empirical measurements, whereas the y-axis represents imputed measurements. (B) Cross-domain ATAC to RNA inference, formatted as in panel (A). (C) ATAC to ATAC intra-domain inference performance. (D) Cross-domain RNA to ATAC inference performance. We observe that all four encoder/decoder combinations perform well. This provides further evidence that BABEL can be trained on data spanning a range of species and experimental protocols.

A

Metric	Joint profiling	Single modality
Fraction reads with valid barcodes	97.8%	97.6%
Percent duplicates	52.3%	27.6%
Estimated number of cells	11,909	8,633
Median fragments per cell	13,486	10,264
TSS enrichment score	9.85	8.52

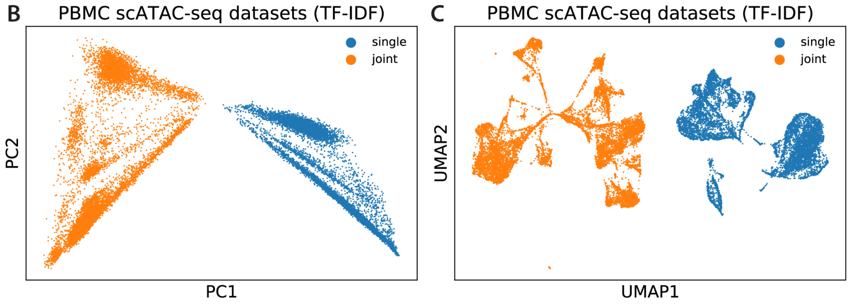

Supplementary Figure S4: Comparison of jointly profiled and single-modality scATAC-seq PBMC data. (A) Summary metrics for these two datasets (taken from 10x data portal), which demonstrate notable differences between datasets. PCA of these two datasets visualize their differences (B). To combine these datasets, we merged peaks that overlap between the two datasets and dropped peaks that do not overlap. This is similar to how we pool datasets for training BABEL. However, rather than binarizing the resultant counts as BABEL does, we apply a TF-IDF transformation prior to performing linear PCA decomposition as shown. While binarizing the data provides modelling advantages, TF-IDF transformations can be more conducive to data visualization. We observe that the first principal component perfectly separates the two different experiments. This indicates that while these two datasets are profiling similar cells with similar summary metrics like TSS enrichment scores, their underlying noise patterns and possibly even some chromatin accessibility patterns, are quantifiably different. (C) shows that this separation between experimental protocols is also seen in non-linear UMAP visualization of the same data. Importantly, we do not perform any data preprocessing to explicitly account for the differences shown here before asking BABEL to perform cross-modality translation.

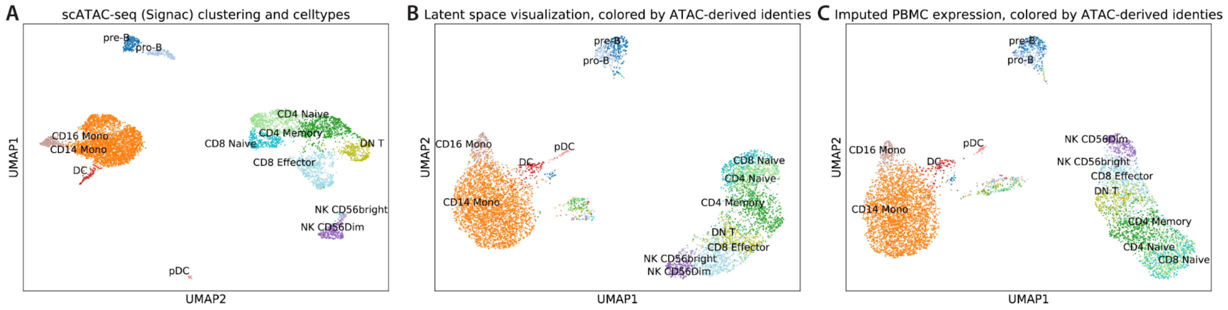

Supplementary Figure S5: Cell type continuity between the three main steps within BABEL's ATAC to expression prediction: ATAC input (A), BABEL latent representation (B), and RNA expression prediction (C). All panels show cells colored by their ATAC-based identities to clearly demonstrate how these are tracked through BABEL. (A) UMAP visualization of the input scATAC-seq human PBMC data, generated via Signac. Axes are swapped for easier visualization with other panels. We see separation between a primarily monocyte cluster (left), a B cell cluster (top), and a CD4/CD8/NK cluster (right). These three main clusters are retained after BABEL projects this scATAC-seq data into its latent representation, visualized in panel (B). This is strong evidence that BABEL's latent space has learned the relationships between cell types, despite having no prior information regarding cell types or even clusters. This latent representation is then used to infer the expression profiles using the RNA decoder, the output of which is visualized in (C). This panel is a duplicate of Figure 3A, reproduced here for ease of comparison, and is very similar to an empirical PBMC scRNA-seq experiment (Figure 3B). We see that throughout all stages in BABEL's ATAC to RNA translation, BABEL preserves the overall biological relationships between these cells.

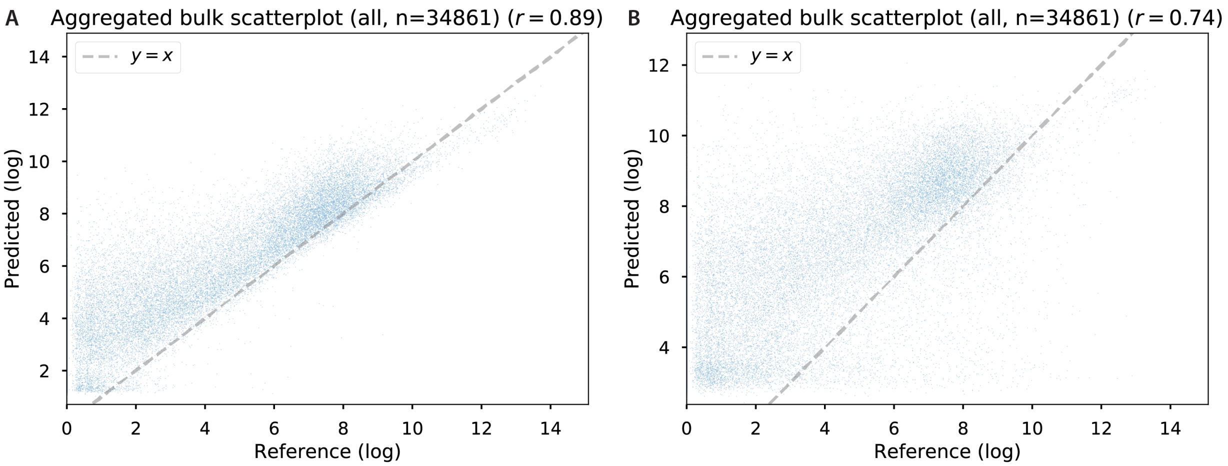

Supplementary Figure S6: Pseudo-bulk correlation of different versions of BABEL on PBMC samples. In both density heatmaps, the x-axis shows measured PBMC scRNA-seq expression, and the y-axis shows BABEL's inferred single-cell expression from PBMC scATAC-seq data. Since the two modalities are not paired, but are from the same tissue, we compute the average expression for each gene (n=34,861) across all cells – the pseudo-bulk expression. (A) Shows pseudo-bulk expression concordance when BABEL is trained on DM, HSR and PBMC data, and (B) shows pseudo-bulk expression concordance when BABEL is trained on just DM and HSR cells, excluding PBMCs. Including PBMCs in the training data improves pseudo-bulk expression correlation with the measured expression. This demonstrates the utility of pseudo-bulk correlation in allowing us to objectively quantify the trustworthiness of BABEL's single-cell predictions even without paired ground truth measurements.

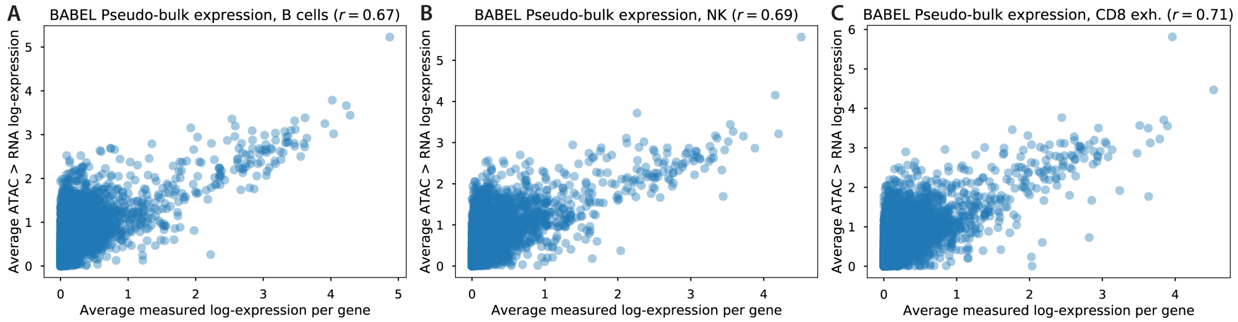

Supplementary Figure S7: Pseudo-bulk correlation of BABEL on BCC samples, broken down by various cell types. X-axis represents expression within a tissue-matched scRNA-seq study averaged across cells; y-axis represents BABEL's single-cell ATAC to RNA predictions, also averaged across cells. Each panel shows pseudo-bulk expression averaged across a different cell type: B cells (A), natural killer cells (B), and CD8 exhausted T cells (C). We find that the pseudo-bulk correlation is consistent for each cell type, not just for the global population of cells (Figure 4A). This provides further evidence that BABEL produces high-quality predictions that are not simply general bulk estimates.

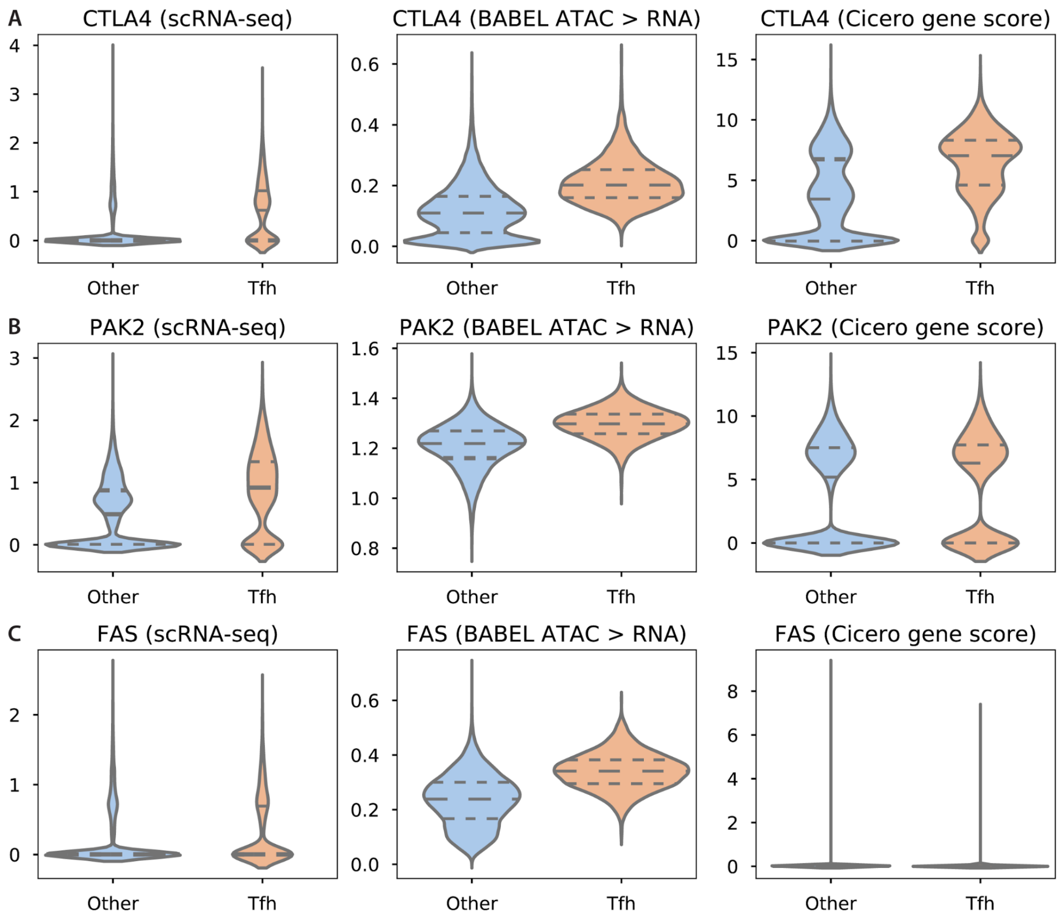

Supplementary Figure S8: Predicted versus empirical overexpression of immunosuppressive genes in Tfh cells. Each gene shown in each row of plots was predicted to be significantly overexpressed by BABEL. All violin plots show expression in log scale, with dashed horizontal lines indicating upper/lower quartiles and median. Since scRNA-seq, BABEL, and Cicero are all normalized differently, absolute numeric values are not comparable. (A) Shows the expression of *CTLA4* within the Tfh cell cluster, compared with remaining cells, as measured by a tissue-matched scRNA-seq experiment (left), as predicted from scATAC-seq data by BABEL (center), and as inferred by Cicero's gene activity scores (right). In this case, both BABEL and Cicero predict overexpression, which is validated by the experimental data ($p < 0.05$, Mann-Whitney test). (B) Similar comparison for *PAK2*, where we see strong overexpression in Tfh cells in empirical scRNA-seq (left panel). BABEL (center) more strongly predicts overexpression here compared to Cicero (right), though both are significant ($p < 0.05$, Mann-Whitney test). (C) Comparison for *FAS*, which exhibits subtle overexpression in scRNA-seq ($p < 0.05$, Mann-Whitney test). Here, BABEL correctly predicts a slight overexpression in the Tfh cluster ($p < 0.05$, Mann-Whitney test), whereas gene activity scores predict no statistically significant overexpression ($p > 0.05$, Mann-Whitney test).

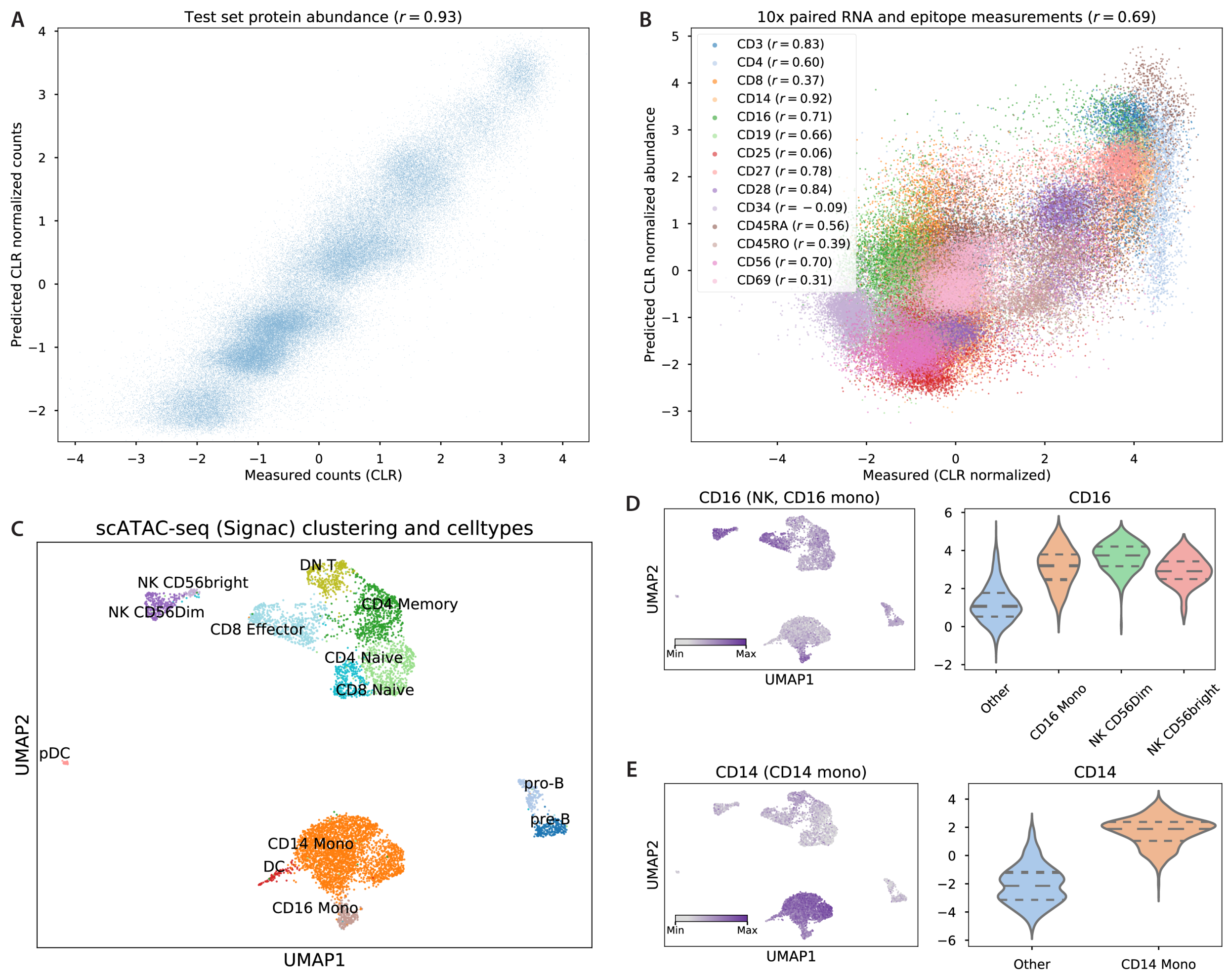

Supplementary Figure S9: Extending BABEL to infer single-cell protein epitopes. Using a set of bone marrow CITE-seq measurements, we train a protein decoder network that takes BABEL's pre-trained latent representation and predicts CLR-normalized protein counts. (A) Shows performance of this approach on a test cluster of 4,032 bone marrow cells, and (B) shows predicted protein expression for 5,221 PBMC cells (also profiled via CITE-seq), colored by protein. Only proteins present in both bone marrow and PBMC CITE-seq measurements are shown. In both panels, the x-axis denotes the CLR-normalized measured protein abundance, the y-axis denotes the protein decoder's predictions given BABEL's latent representation of these cells' expression signatures. Each point represents a single cell's abundance of a given protein. In both cases, we see that BABEL can be successfully extended to predict epitope measurements from expression profiles (Pearson's correlations of 0.93 and 0.69, respectively), even showing strong correlation within each protein (B). (C-E) show that we can use this protein decoder to predict epitopes from scATAC-seq, even without training this specific modality pair. (C) Visualizes a PBMC scATAC-seq dataset (reproduction of Supplementary Figure S5A). We use these chromatin accessibility profiles to impute epitopes. (D) Predicted abundance of CD16 for each cell. CD16 is a marker associated with CD16 monocytes and NK cells – a pattern that is recapitulated by BABEL's ATAC to protein predictions (violin plot, right panel). (E) Predictions for CD14, which BABEL correctly predicts to be most abundant in CD14 monocytes.

Supplementary Note

Chromosome-aware ATAC encoder/decoder architecture

BABEL's component networks that encode and decode ATAC chromatin accessibility data are designed to leverage the intuition that most chromatin interactions occur on an intra-chromosomal level(31). Accordingly, we prune many of the inter-chromosomal parameters in BABEL's ATAC encoder and decoder networks. In the following, we provide an estimate for how many parameters this approach saves. We describe the ATAC decoder, but the analysis is the same for the encoder.

For simplicity, we only consider parameters directly involved in linear matrix transformations, disregarding parameters needed for activation functions, batch normalization, etc. When trained on human data, BABEL learns to predict 223,897 peaks across 22 autosomes; we simplify this as 10,000 peaks per chromosome. Recall that the shared latent space is 16-dimensional, and maps to a concatenated layer of 16 dimensions per chromosome. Thus, the latent to "concatenated" layer contains 16 x (22 x 16) = 5,632 parameters; this is constant whether we are using chromosome-specific architectures or not, and allows for limited representation of inter-chromosomal interactions in the former case. Under the chromosome-aware design, this then leads to 22 chromosome sub-units, mapping from 16 to 32 to 10,000 output dimensions: 22 x (16 x 32 + 32 x 10000) = 7,051,264 weights. Under the chromosome-agnostic design, we would instead have (22 x 16) x (22 x 32) + (22 x 32) x 220000 = 155,127,808 weights, or 22 times the number of weights for the final two layers.

BABEL's composition of encoder and decoder networks and their shared latent representation

BABEL takes the approach of composing two encoder and two decoder neural networks, such that the encoder and decoder networks are interoperable. We benchmarked this approach against a naive approach that learns four completely separate neural networks with no interoperability constraints. These four neural networks are architecturally identical to each of BABEL's four encoder/decoder combinations and is each trained to only perform RNA to RNA, RNA to ATAC, ATAC to RNA, or ATAC to ATAC prediction. Compared to BABEL, this naive approach uses twice the number of overall parameters, as it requires 4 separate pairs of decoders and encoders compared to BABEL's combinatorial use of 2 encoders and 2 decoders.

We performed our evaluation using the GM12878 paired data, as this dataset is fully external to model training and is thus the most stringent measure of model generalizability and performance. We found that for all tasks, BABEL either matches or outperforms these independent models (Supplementary Table S2). This suggests that our interoperability constraint has no overall negative impact on performance, and even seems to help BABEL learn more general representations and functions for translating cellular modalities. Furthermore, these results confirm the efficacy of our enforcement of a shared latent representation via loss-induced encoder-decoder interoperability. In contrast, prior works translating between multi-omic profiles did not leverage paired measurements, and consequently required complex adversarial networks or expensive manifold alignment strategies to align points in a similar shared latent representation(15–17). Since BABEL's latent representation is already aligned (otherwise BABEL's

performance wouldn't be able to match/exceed that of single-purpose dedicated models), such manifold alignments approaches would only add unnecessary complexity.

Beyond being a well aligned shared representation, BABEL's latent representation serves as a critical information bottleneck as well. Namely, by restricting the size and thus possible information content of the latent representation that all encoder/decoder combinations must use, we restrict our model to focusing on the most important factors driving cell-to-cell variation. This provides implicit regularization and is similar to how tools like principal component analysis can denoise data by compressing data into the top few principal components. When designing BABEL, we considered several sizes for this latent representation. We found that increasing the dimensionality of the latent space from 16 to 32 or even 64 provided no consistent benefit in BABEL's ability to perform well on the test set (Supplementary Table S1). In the absence of any meaningful performance impact, we elected to use the most restrictive latent representation of 16 dimensions to minimize potential model overfit.

Furthermore, we see evidence that BABEL's 16-dimensional latent space encodes biologically meaningful relationships. If we apply BABEL's ATAC encoder network to scATAC-seq PBMC data (Supplementary Figure S5A) and visualize the resultant latent representation using UMAP, coloring each point by its cell type, the resulting visualization (Supplementary Figure S5B) is highly similar to reference plots generated by applying UMAP to empirical scATAC-seq and scRNA-seq (Figure 3B). Remarkably, not only are cells of the same cell type grouped together in the latent representation, but similar cell types also appear to be closer in this latent space as well. For example, CD14 and CD16 monocytes occupy similar (but distinct) regions in the latent space, which is reflective of their biological similarity compared to other PBMC cell types; the same can be said of pro-B/pre-B cells, natural killer subtypes, etc. This suggests that BABEL's latent space effectively captures important biological variation, even without being given prior information regarding cell type identities or relationships. In fact, we suspect that this "biologically continuous" representation improves generalization, as it allows for efficient interpolated representation across a spectrum of intermediate cell types within the latent space. This is especially evident compared to a hypothetical alternative where the latent space naively represents each cell type independently of other cell types; this would necessitate a great amount of training data to learn every individual cell type via rote memorization and would likely struggle to generalize to even slight variations on previous data.

Understanding BABEL's performance on PBMCs through data ablation

We hypothesize that one of the key factors driving BABEL accuracy in predicting PBMC cells' single-cell gene expression is the inclusion of PBMCs in its training data. To quantitatively measure this in the absence of paired measurements, we compare pseudo-bulk expression signatures (i.e., the mean across cells of every gene's expression). We observe that BABEL's imputed pseudo-bulk PBMC expression exhibits a Pearson's correlation of 0.89 when compared to a pseudo-bulk derived from the aforementioned PBMC scRNA-seq experiment (Supplementary Figure S6A). This correlation is highly consistent across different versions of BABEL trained using different cross-validation folds (Supplementary Table S5). However, if we remove PBMCs from BABEL's training set and re-train BABEL from scratch, this pseudo-bulk correlation drops to 0.74 (Supplementary Figure S6B). This analysis concretely demonstrates the benefits of training

BABEL on a similar group of cells, and also highlights how aggregate metrics may be used to quantify BABEL's performance in a general setting without paired measurements.
